# Targeting focal adhesion kinase boosts immune response in *KRAS/LKB1* co-mutated lung adenocarcinoma via remodeling the tumor microenvironment

**DOI:** 10.1186/s40164-023-00471-6

**Published:** 2024-01-30

**Authors:** Meng Qiao, Fei Zhou, Xinyu Liu, Tao Jiang, Haowei Wang, Xuefei Li, Chao Zhao, Lei Cheng, Xiaoxia Chen, Shengxiang Ren, Zaiqi Wang, Caicun Zhou

**Affiliations:** 1 Department of Medical Oncology, Shanghai Pulmonary Hospital & Thoracic Cancer Institute, Tongji University School of Medicine, No. 507, Zheng Min Road, Shanghai, 200433 People’s Republic of China; 2 Department of Medical Oncology, Ruijin Hospital, Shanghai Jiao Tong University School of Medicine, Shanghai, 200025 People’s Republic of China; 3 Department of Lung Cancer and Immunology, Shanghai Pulmonary Hospital, Tongji University School of Medicine, Shanghai, 200433 People’s Republic of China; 4 InxMed (Shanghai) Co., Ltd, Shanghai, 201202 People’s Republic of China

**Keywords:** Focal adhesion kinase, KRAS, LKB1, Drug resistance, Tumor microenvironment

## Abstract

**Background:**

*KRAS* mutation is one of the most common oncogenic drivers in NSCLC, however, the response to immunotherapy is heterogeneous owing to the distinct co-occurring genomic alterations. *KRAS/LKB1* co-mutated lung adenocarcinoma displays poor response to PD-1 blockade whereas the mechanism remains undetermined.

**Methods:**

We explored the specific characteristics of tumor microenvironment (TME) in KL tumors using syngeneic *KRAS*^*G12D*^*LKB1*^*−/−*^ (KL) and *KRAS*^*G12D*^*TP53*^*−/−*^ (KP) lung cancer mouse models. The impact of focal adhesion kinase (FAK) inhibitor on KL lung tumors was investigated in vitro and in vivo through evaluation of both KL cell lines and KL lung cancer mouse models.

**Results:**

We identified KL tumors as “immune-cold” tumors with excessive extracellular matrix (ECM) collagen deposition that formed a physical barrier to block the infiltration of CD8^+^T cells. Mechanistically, abundant activated cancer-associated fibroblasts (CAFs) resulted from FAK activation contributed to the formation of the unique TME of KL tumors. FAK inhibition with a small molecular inhibitor could remodel the TME by inhibiting CAFs activation, decreasing collagen deposition and further facilitating the infiltration of anti-tumor immune cells, including CD8^+^ T cells, DC cells and M1-like macrophages into tumors, hence, converting “immune-cold” KL tumors into “immune-hot” tumors. The combined FAK inhibitor and PD-1 blockade therapy synergistically retarded primary and metastatic tumor growth of KL tumors.

**Conclusions:**

Our study identified FAK as a promising intervention target for KL tumors and provided basis for the combination of FAK inhibitor with PD-1 blockade in the management of KL lung cancers.

**Supplementary Information:**

The online version contains supplementary material available at 10.1186/s40164-023-00471-6.

## Background

*KRAS* mutation is one of the most common oncogenic drivers in non-small cell lung cancer (NSCLC), accounting for ~ 25% of Caucasians and ~ 10% of Asians [1, 2]. Recently, immune checkpoint inhibitors (ICIs) that target programmed cell death protein-1 (PD-1)/PD ligand-1 (PD-L1) axis have significantly prolonged survival outcomes of patients with metastatic NSCLC, especially for patients with KRAS mutations [3].

Notably, not all patients with *KRAS* mutations benefit from ICIs, largely because of tumor heterogeneity-a consequence of co-occurring genomic alterations. Skoulidis et al. divided *KRAS*-mutant lung adenocarcinoma into three subgroups on account of concomitant co-mutations, namely *STK11/LKB1* mutations (KL subgroup), *TP53* mutations (KP subgroup), and (KRAS-only subgroup) and revealed that KL subgroup responded poorly to ICIs, with an objective response rate (ORR) of only 7.4% [4]. As *STK11/LKB1* mutations occur in approximately one-third of *KRAS*-mutated lung adenocarcinomas [5], to uncover the potential mechanism is in high demand and is critical for developing effective combination strategies.

Several lines of evidence have demonstrated that loss of LKB1 could (i) up-regulate c-MYC expression by promoting MZF1 transcription and increase the secretion of IL-23 and CCL9, hence, inhibit the recruitment of CD3^+^ T cells [6]; (ii) activate the WNT/β-catenin pathway in tumors cell to promote CCL4 production and thereby inhibiting recruitment of CD103^+^ dendritic cells (DCs) [7]; (iii) increase the expression of IL-6 which can recruit more tumor associated neutrophils (TANs) [8]; and (iv) silence STING transcription to result in insensitivity to cytoplasmic double-strand DNA detection, further facilitating immune escape [9]. All these findings, in view of intrinsic features of tumor cells or immune cells, partially explain why KL tumors develop primary resistance to ICI monotherapy. Of note, KL tumors were also refractory to combination of ICIs and chemotherapy [10]. Therefore, other important components in the tumor microenvironment (TME), particularly extracellular matrix (ECM) and cancer-associated fibroblasts (CAFs) which are also now recognized to play essential roles in antitumor immunity raised our research interest [11]. Therapeutic targeting of ECM or CAFs have recently represented a promising strategy in the anticancer arsenal. Intriguingly, a previous study demonstrated that KL lung cancer model had high levels of collagen deposition compared to *KRAS*-only model, while the mechanistic explanation was not clearly illustrated [12]. Considering the pivotal role of ECM and CAFs in TME composition, it is of significance to unravel the potential mechanism of their impact on anti-tumor immunity.

Here, using syngeneic *KRAS*^*G12D*^*LKB1*^*−/−*^ lung cancer mouse models, our study unveiled pivotal roles of ECM and CAFs in the formation of “immune-cold” KL tumors. We found that more ECM collagen deposited in KL tumors compared with KP tumors, resulting in forming a physical barrier to inhibit the infiltration of CD8^+^T cells, mechanistically owing to abundant CAFs with fibroblastic focal adhesion kinase (FAK) activation in the TME. Furthermore, using a novel FAK inhibitor (IN10018, received fast track designation from the U.S. Food and Drug Administration and Breakthrough Therapy Designation by the China National Medical Products Administration) to inhibit the activation of CAFs could break the physical barrier, effectively recruit effector immune cells into the TME, thus boosting anticancer immune response in KL lung cancer mouse models. Importantly, the combination of FAK inhibitor and PD-1 blockade could significantly retard primary and metastatic tumor growth of KL tumors.

## Methods

### Human data sharing

The OAK and POPLAR trials were available as supplementary information in Gandara DR et al. The MSKCC studies are available at www.cbioportal.org. The patient survival data and genetic aberrations were extracted for subgroup analysis.

### Cell lines and reagents

Mouse KL cell line was a gift of Ji et al. Somatic *KRAS* activation and *Lkb1* loss were induced in the lungs of genetically engineered male mice (*Kras*^*G12D/*+^*/Lkb1*^*L/L*^ with the C57BL6/J genetic background) by nasal inhalation of Adeno-Cre (2 × 10^6^ PFU). After 12 weeks Adeno-Cre treatment, tumors from mice of defined genotypes were dissected and cut into small pieces and cultured in RPMI-1640 + 10% FBS + 1% penicillin–streptomycin. The medium was changed every other day until cells outgrew and stable KL cell lines formed. Mouse KP cell line was provided by InxMed. A549 and H1299 cell lines were purchased from American Type Culture Collection (ATCC). L929 cell line was kindly provided by Stem Cell Bank, Chinese Academy of Sciences. Cells were maintained in DMEM or RPMI1640 (HyClone) supplemented with 10% fetal bovine serum (FBS) (Gibco) and 1% penicillin–streptomycin (Gibco). The small molecule FAK inhibitor IN10018 was provided by InxMed. Anti-PD-1 mAb (#BE0146, clone: RMP1-14), IgG control (#BE0089, clone: 2A3), InVivoPure™ pH 7.0 Dilution Buffer (#IP0070) and InVivoPure™ pH 6.5 Dilution Buffer (#IP0065) were purchase from BioXcell (West Lebanon, New Hampshire, USA).

### Plasmid and siRNA transfection

Human-STK11 and mouse-STK11 were generated by PCR, cloned into pHBLV-vector and transfected into cells using lipofectamine3000. siRNAs were commercially synthesized (Ribo-Bio Co) and transfected into cells using riboFECT CP Transfection Kit (Ribo-Bio Co). siRNA sequences targeting human genes were as below: siSTK11-1, TGAGGAGGTTACGGCACAA.

### Quantitative RT-PCR (qRT-PCR)

Total RNAs were isolated from cells using RNAiso Plus (TAKARA) and reverse-transcribed to cDNA using RevertAid First Strand cDNA Synthesis Kit (Thermo Scientific). Diluted cDNA was subject to qRT-PCR analysis using TB Green™ Premix Ex Taq™ II (TAKARA). Relative expression levels were calculated by the comparative Ct approach. qRT-PCR primers were listed as follows: m-FAP-Forward, 5′-GTCACCTGATCGGCAATTTGT-3′ and m-FAP-Reverse, 5′- CCCCATTCTGAAGGTCGTAGAT-3′; m-actin-Forward, 5′- TCCTGTGGCATCCACGAAACTACA-3′ and m-actin-Reverse, 5′- ACCAGACAGCACTGTGTTGGCATA-3′.

### Western blotting

Protein samples were extracted from cells using RIPA lysis buffer (Beyotime). The quantification of protein level was measured with BCA kit (Beyotime). The samples were boiled in 5X loading buffer, separated by SDS-PAGE, transferred to PVDF membranes (Millipore). After blocking, the membrane was incubated overnight at 4 °C with primary antibodies as followings: LKB1 (#3047, Cell Signaling Technology; 1:1000 dilution), FAK (#610,087, BD Pharmingen; 1:1000 dilution), p-FAK (#81,298, Abcam; 1:1000 dilution), GAPDH (#5174, Cell Signaling Technology; 1:1000 dilution). The membrane was then washed with TBST and incubated with secondary antibody for 1 h at room temperature in the dark. The targeted proteins were detected by ECL reagent (Tanon) and the exposure was performed by ChemiDoc XRS (Bio-Rad).

### Cell counting kit-8 (CCK-8) assay

KL cells were split into 96-well plate (3 × 10^3^ cells, 100 μL per well, six replicates). The culture medium was changed into medium with different dose of FAK inhibitor on the other day. After incubating for 24 h, 48 h and 72 h, 10 μL of the CCK-8 (Dojindo) was added to each well of the plate and continued to incubate for 2 h. Absorbance was measured at a wavelength of 450 nm with microplate reader (Bio-Tek).

### Cell apoptosis assay

The rates of cell apoptosis were measured by an Annexin V-FITC/PI Apoptosis Detection Kit (Yeasen biotech Co., Ltd.). Briefly, cells were harvested and washed with cold PBS twice. After being resuspended in 100 μL of binding buffer, cells were incubated with Annexin V and PI for 15 min in the dark. Then the apoptosis data was acquired on Beckman Cytoflex (Beckman Coulter). The results were analyzed with FlowJo 10.6.1 software (TreeStar).

### RNA-seq and data analysis

The mouse tumor tissue was harvested for total RNA extraction with TRIzol (Invitrogen). RNA integrity was assessed using the RNA Nano 6000 Assay Kit of the Bioanalyzer 2100 system (Agilent Technologies). A total amount of 1 μg RNA per sample was used as input material for the RNA sample preparations. The cDNA libraries were generated with NEBNext Ultra RNA Library Prep Kit for Illumina (New England Biolabs Inc). The clustering of the index-coded samples was performed on a cBot Cluster Generation System using TruSeq PE Cluster Kit v3-cBot-HS (Illumia) according to the manufacturer’s instructions. After cluster generation, the library preparations were sequenced on an Illumina Novaseq platform and 150 bp paired-end reads were generated. Gene expression levels were quantified as normalized FPKM (fragments per kilobase of exon per million mapped fragments). GSEA was performed using local version of the GSEA analysis tool (http://www.broadinstitute.org/gsea/index.jsp). KEGG pathways and GO enrichment analysis of differentially expressed genes were implemented by clusterProfiler R package. The Reactome database brings together the various reactions and biological pathways of human model species. The corrected P value less than 0.05 were considered significantly enriched by differential expressed genes in Reactome database. Heatmaps were produced by Novomagic (https://magic.novogene.com). FPKM RNA sequencing data was uploaded to GEO (GSE244452).

### In vivo tumor formation assay

A total of 2 × 10^6^ KP or KL cells were suspended in 200 μL phosphate-buffered saline (PBS) and then injected subcutaneously into 6 week old male C57BL/6 mice in the right flank. The tumor volumes were measured by caliper and calculated with a formula of π/6 × long diameter × short diameter × short diameter. Mice were sacrificed when tumor volume reached 700–800 mm^3^. Tumors from KP or KL cell-bearing mice were excised for further analysis.

### In vivo drug response and tumor growth assays

To explore the efficacy of PD-1 inhibitor in KP and KL mouse model, 2 × 10^6^ KP or KL cells were suspended in 200 μL PBS and were injected subcutaneously in the right flank of syngeneic recipient 6–8 week old male mice. Mice bearing tumors between 150 and 200 mm^3^ were randomly assigned to intraperitoneal treatment with six doses of 200 μg anti-PD-1 (clone RMPI-14; BioXCell) or isotype control antibody (clone 2A3; BioXCell) administered every 3 days (N = 5–8 mice per group). The body weights and tumor volumes were monitored and recorded twice a week.

To explore the impact of FAK inhibitor and combination therapy in KL mouse models, 2 × 10^6^ KL cells were suspended in 200 μL PBS and were injected subcutaneously in the right flank of syngeneic recipient male mice. FAK inhibitor treatment or vehicle control reagent (0.5% Natrosol 250 HX dissolved in distilled water) was initiated at the first day when tumor volume reached at indicated size and administered at 25 mg/kg by oral gavage every day. Anti-PD-1 (clone RMPI-14; BioXCell) or isotype control antibody (clone 2A3; BioXCell) were administered at 200μg/mouse in total of six doses intraperitoneally. Tumor volume was measured with a formula of 0.5 × long diameter × short diameter × short diameter. At indicated day, tumor tissues or spleen were harvested for analysis.

All the animal studies were approved by the Institutional Committee for Animal Care and Use, Tongji University School of Medicine, and were performed in accordance with the institutional guidelines.

### Histologic analysis

4 μm paraffin-embedded tissue were deparaffinized and rehydrated, stained with hematoxyline and eosin (H&E), IHC, IF, Masson's Trichrome Kit (SenBeiJia Biological Technology Co., Ltd.) and Picro-Sirius Red (SenBeiJia Biological Technology Co., Ltd.) following manufacturer protocol. For IHC, slides were incubated overnight in a humidified chamber at 4 °C with primary antibodies as followings: LKB1 (#13,031, Cell Signaling Technology; 1:250 dilution), Collagen I (#BA0325, Boster; 1:250 dilution), Collagen III (#ab7778, Abcam; 1:200 dilution), CD8 (#98,941, Cell Signaling Technology; 1:400 dilution), HIF-1α (#ab114977, Abcam; 1:250 dilution), p-FAK (Tyr397) (#44624G, Invitrogen; 1:200 dilution). The whole slides were scanned with Pannoramic 250 (3D HISTECH). The intensities of brown-colored precipitate stained with DAB were measured and quantified with IOD or cell intensity by Image Pro Plus 6 (Media Cybernetics). For IF, the FAP/α-SMA was used for activated CAF analysis (FAP: #ab53066, Abcam, 1:200 dilution; α-SMA: #ab7817, Abcam, 1:1000 dilution). CD31 (#77,699, Cell Signaling Technology; 1:100 dilution) was used for vascular analysis. The whole slides were scanned with Pannoramic MIDI (3D HISTECH).

### Flow cytometry

The single-cell suspensions of tumor tissue were generated with tumor dissociation kit, mouse (Miltenyi Biotec) according to manufacturer’s instructions and mouse spleen were mechanically dissociated. After re-suspended in FACS buffer (PBS, 2%FBS), the cells were blocked with anti-mouse CD16/CD32 (eBioscience) 10 min prior to staining with surface antibodies at 4 °C. Surface markers were then added along with 10 μL Brilliant Stain Buffer (BD Bioscience) in a final staining volume to 100 μL. For intracellular staining, cells were fixed and permeabilized with Transcription Factor Buffer Set (BD Bioscience) followed by staining with intracellular antibodies. Fluorochrome-conjugated antibodies for flow cytometry analysis were listed in Additional file 1: Table S1. The flow cytometry data was acquired on Beckman Cytoflex (Beckman Coulter) and the results were analyzed with FlowJo 10.6.1 software (TreeStar).

### Cytokine assays

The intratumoral CCL2, CCL5, CCL7, CXCL1, CXCL2 and VEGF were measured using Luminex liquid suspension chip. Mouse Premixed Multi-Analyte Kit (#LXSAMSM-06, R&D systems) was used in accordance with manufacturer’s instructions. Briefly, the lysate of tumor tissue was embedded with microbeads for 2 h, and then incubated with detection antibody for 1 h. Subsequently, streptavidin-PE was added into each well for 30 min, and values were read using the Bio-Plex MAGPIX System (Bio-Rad). The intratumoral CXCL9 (#MCX900, R&D systems) and CXCL5 (#ab100719, Abcam) were analyzed with commercial ELISA Kit according to the manufacturer's instructions.

### Statistical analysis

Results were presented as mean ± SEM. The log-rank test was used for comparison of survival outcomes downloaded from OAK, POPLAR and MSKCC with Kaplan–Meier method. Unpaired Student’s *t*-test was used to compare the statistical significance between tested and control groups. For comparison of tumor growth between two groups in vivo, two-way analysis of variance (ANOVA) with Tukey’s post hoc test was performed (control versus FAK inhibitor group in Fig. 6A; control versus anti-PD1 and FAK inhibitor versus FAK inhibitor + anti-PD1 group in Fig. 7C) [13].Survival time of mice was defined from the day of tumor cell inoculation until the tumor volume reached 1000 mm^3^.Statistical analyses were performed using GraphPad Prism 8 (GraphPad Software). Statistical significance was determined as indicated in the figure legends. Significance was set to P < 0.05 and represented as *P < 0.05, **P < 0.01, ***P < 0.001.

## Results

### KL lung adenocarcinoma displayed a poor response to ICI therapy

**Fig. 1:**
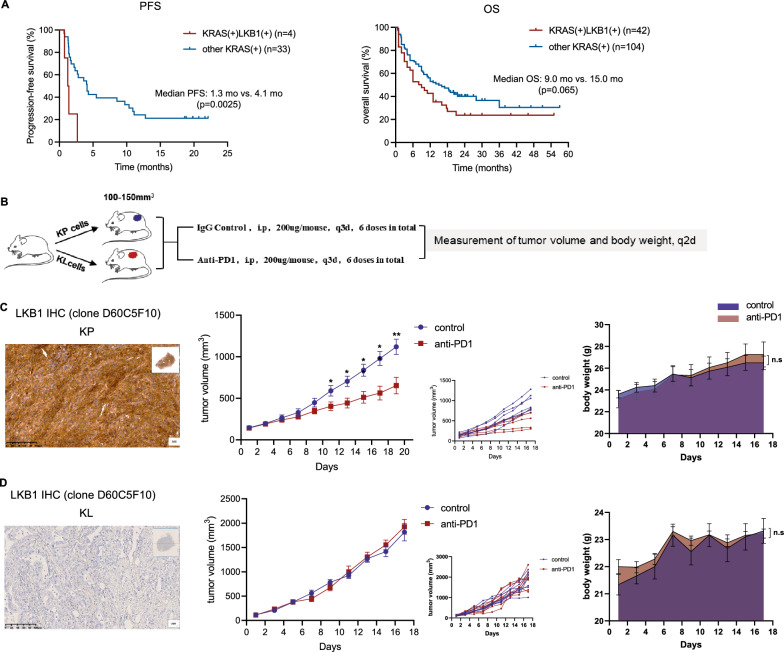
The efficacy of immunotherapy in KL and KP tumors. **A** Survival data extracted from public database. PFS data was extracted from OAK and POPLAR and OS data was extracted from OAK, POPLAR and MSKCC. The log-rank test was used for comparison of survival outcomes with Kaplan–Meier method. p values are shown. **B** Diagram depicting treatment schedule for subcutaneous KP and KL tumor models. **C**, **D** Identification of LKB1 expression, tumor volume change and body weight of subcutaneously implanted KP or KL tumors in treatment of control or anti-PD1 group (n = 5–8 per group). Statistical analysis was performed using unpaired Student’s *t*-test. *p < 0.05, **p < 0.01, ***p < 0.001, and ^ns^p-values with no statistical difference. KL, *KRAS*^*G12D*^*LKB1*^*−/−*^; KP, *KRAS*^*G12D*^*TP53*^*−/−*^; PFS, progression-free survival; OS, overall-survival; LKB1, liver kinase B1; PD1, programmed death-1

Firstly, we investigated the impact of *KRAS/LKB1* co-mutations on survival outcomes in PD-1/PD-L1 inhibitor-treated lung adenocarcinoma patients from POPLAR and OAK trials and confirmed that KL subgroup had significantly shorter progression-free survival (PFS) compared with other *KRAS*-mutated patients (median PFS:1.3 vs. 4.1 months, p = 0.0025). Integrating the data from MSKCC cohort, we further demonstrated that overall survival (OS) also tended to be shorter in KL group (median OS: 9.0 vs. 15.0 months, p = 0.065) (Fig. 1A).

Previous studies reported that among all subtypes of *KRAS*-driven lung cancers, tumors with frequently inactivated TP53 were most sensitive to PD-1 blockade [4]. Therefore, to explore the underlying mechanisms for the distinct therapeutic response, we used *KRAS*^G12D^*LKB1*^−/−^ (hereafter referred to “KL”, previously established from a spontaneously arising lung adenocarcinoma in the *Kras*^G12D^*Lkb1*^fl/fl^ mouse model, (Additional file 3: Fig. S1A) and *KRAS*^G12D^*TP53*^−/−^ (hereafter referred to “KP”) murine lung adenocarcinoma cell lines [14, 15] for further analyses. Isogenic cell lines upon confirmation of *STK11* knockout (by immunoblotting for the LKB1 protein) (Additional file 3: Fig. S1B) were implanted into the right flank of syngeneic recipient mice and cohorts of tumor-bearing mice were randomized to be treated with anti-PD-1 antibody or IgG control (Fig. 1B). The expression of LKB1 by IHC in KP or KL tumors further confirmed that *STK11* was successfully knocked out (Fig. 1C and D). Tumor growth curve suggested that anti-PD-1 antibody significantly attenuated tumor growth in subcutaneous KP mouse model (Fig. 1C) but showed no efficacy in KL mouse model (Fig. 1D). No significant differences in body weight were found between anti-PD-1 antibody and control group in KP or KL mouse model. These results suggested that KL tumors were resistant to anti-PD1 antibody and our KL mouse model was reliable for subsequent research.

### KL tumors revealed immunosuppressive TME with fewer immune-active cells and more immunosuppressive cells infiltration

**Fig. 2:**
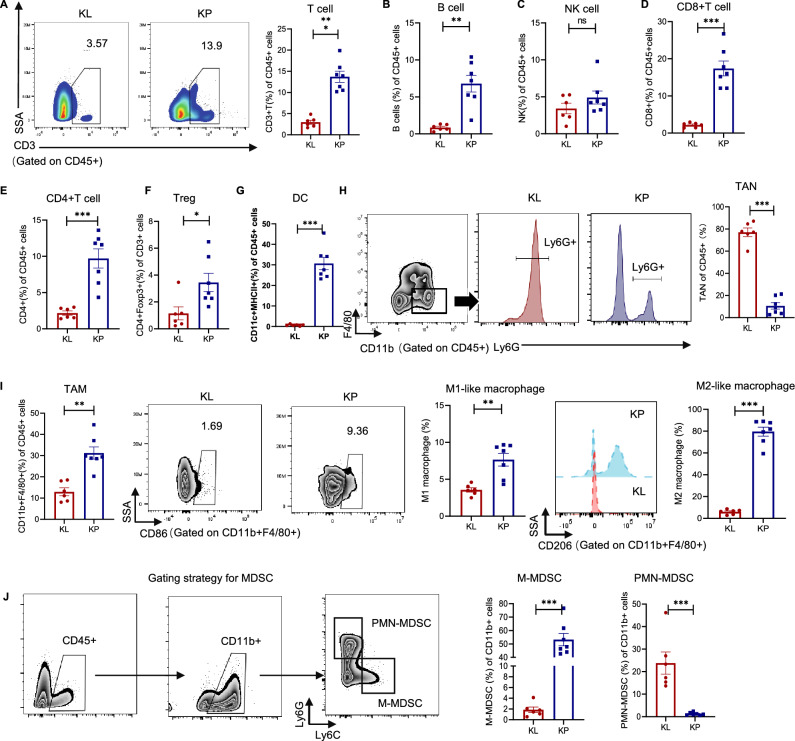
KL tumors displayed immunosuppressive TME with fewer immune-active cells and more immunosuppressive cells infiltration. Subcutaneous KP and KL tumors were dissected for flow cytometry analysis when tumor volume reached 700–800 mm^3^ (n = 6 tumors in KL group, n = 7 tumors in KP group). **A** Representative flow cytometry images and histogram showed the percentage of CD3^+^ of CD45^+^ cells in each group. **B** Ratio of CD19^+^ to CD45^+^ cells. **C** Ratio of CD3^−^NK1.1^+^ to CD45^+^ cells. **D** Ratio of CD8^+^ of CD45^+^ cells. **E** Ratio of CD4^+^ to CD45^+^ cells. **F** Ratio of CD4^+^Foxp3^+^ to CD3^+^ cells. **G** Ratio of CD11c^+^MHCII^+^ of CD45^+^ cells. **H** Representative flow cytometry images and histogram showed the percentage of CD11b^+^F4/80^−^Ly6G^+^ of CD45^+^ cells. **I** Representative flow cytometry images and histogram showed the percentage of macrophage (CD11b^+^F4/80^+^ of CD45^+^cells), M1-like macrophage (CD86^+^) and M2-like macrophage (CD206^+^). **J** Gating strategy for MDSCs and histogram showed the percentage of M-MDSC (Ly6C^+^Ly6G^−^) and PMN-MDSC (Ly6G^+^Ly6C^−^). Results in each group were presented as mean ± SEM. *p < 0.05, **p < 0.01, ***p < 0.001, and ^ns^p-values with no statistical difference. KL, *KRAS*^*G12D*^*LKB1*^*−/−*^; KP, *KRAS*^*G12D*^*TP53*^*−/−*^; TME, tumor microenvironment; NK, nature killer; MHC, major histocompatibility complex; Foxp3, forkhead box P3; M-MDSC, monocytic-myeloid-derived suppressor cells; PMN-MDSC, polymorphonucler-myeloid derived suppressor cells; SEM, standard error of mean

We next sought to elucidate how LKB1-deficiency impacted the TME. Firstly, comprehensive immune cell populations between KP and KL tumors via flow cytometry analysis were compared. In general, the infiltrated quantity of T cells (CD3) and B cells (CD19) were significantly decreased in KL tumors as compared with KP tumors (Fig. 2A and B) while no significant difference was observed regarding NK cells (CD3^−^NK1.1^+^) (Fig. 2C). Moreover, KL tumors had significantly fewer CD8^+^T cells, CD4^+^T cells and Foxp3^+^Tregs infiltration (Fig. 2D–F). In addition, DCs (CD45^+^CD11c^+^MHC-II^+^) and tumor-associated macrophage (TAMs)[16], regardless of M1-like macrophage (CD11b^+^F4/80^+^CD86^+^) or M2-like macrophage (CD11b^+^F4/80^+^CD206^+^) were also less infiltrated in KL tumors than KP tumors (Fig. 2G and I). Tumor-associated neutrophils (TANs) and polymorphonuclear myeloid-derived suppressor cells (PMN-MDSCs, CD45^+^CD11b^+^Ly6G^+^Ly6C^low^), which suppressed T cells function to help form immunosuppressive TME [17], were significantly highly infiltrated in KL tumors (Fig. 2H and J). Taken together, our data demonstrated that KL tumors revealed immunosuppressive TME, with fewer immune-active cells infiltration but with more immunosuppressive cells infiltration, including PMN-MDSC and TANs.

### KL tumors had excessive ECM deposition and collagen, which block T cells infiltration into tumor nest

**Fig. 3:**
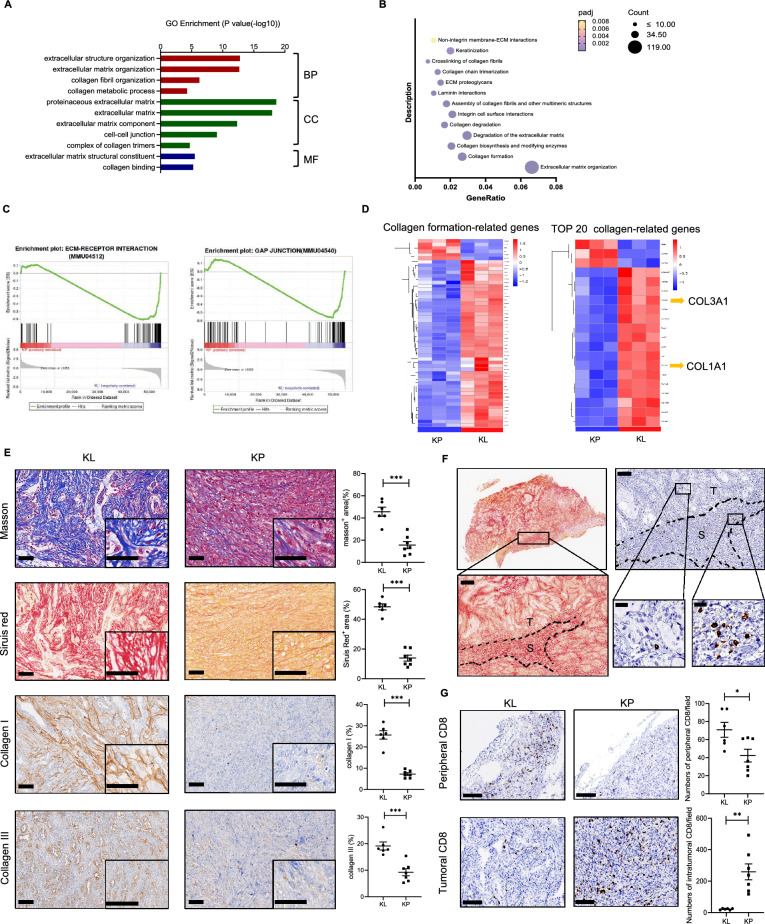
KL tumors had excessive collagen deposition and collagen blocks T cell infiltration into tumor nest. **A** GO analysis for significantly upregulated genes in KL tumors compared to KP tumors. Representative and related GO signal pathways among top 30 upregulated pathways were listed here. **B** Reactome analysis for significantly upregulated genes in KL tumors compared to KP tumors. Representative and related Reactome signal pathways among top 20 upregulated pathways were listed here. **C** GSEA analysis for ECM-receptor interaction and GAP junction in KL and KP tumors. **D** Heatmap of RNA-seq showing collagen-related genes statistically significant (FDR < 0.05) differentially expressed in KL subcutaneous tumors. **E** Representative images and quantification of area performed by Masson, Siruis Red, Collagen I and Collagen III. Unpaired Student’s t-test was used to compare the statistical significance between two groups. Scale bars, 100 μm. inset scale bars, 50 μm. **F** Representative images showing spatial relations between collagen deposition and CD8^+^TILs infiltration. Scale bars, 100 μm. inset scale bars, 20 μm. **G** Representative images and quantification of CD8^+^TILs infiltrated in tumor nest and stroma in two groups. Scale bars, 100 μm. Results in each group were presented as mean ± SEM. *p < 0.05, **p < 0.01, ***p < 0.001, and ^ns^p-values with no statistical difference. KL, *KRAS*^*G12D*^*LKB1*^*−/−*^; KP, *KRAS*^*G12D*^*TP53*^*−/−*^; BP, biological process; CC, cellular component; MF, molecular function; ECM, extracelluar matrix; TIL, tumor infiltrating lymphocyte; SEM, standard error of mean

To uncover the mechanisms that contributed to the formation of immunosuppressive TME of KL tumors, RNA-seq-based transcriptome profiling of KL and KP subcutaneous tumors was analyzed. Briefly, 3497 genes were up-regulated and 3952 genes were down-regulated in KL tumors compared with KP tumors. Furthermore, GO, Reactome and gene set enrichment analysis (GSEA) were performed and showed that genes encoding for ECM-related components were significantly enriched (Fig. 3A–C). The ECM provided a physical scaffold for all cells in the TME and is the major component. Collagen has been implicated in cancer progression, metastasis, and drug resistance. As expected, the transcriptomic data showed that collagen-formation related genes were mostly upregulated in KL tumors. Among top 20 collagen-related genes, COL3A1 and COL1A1 were significantly upregulated in KL tumors (Fig. 3D, Additional file 2: Table S2). Consistently, Sirius Red and Masson staining confirmed the increased intra-tumoral collagen deposition and specifically, collagen I and III, which are encoded by COL1A1 and COL3A1, in KL tumors (Fig. 3E ). To elucidate the impact of collagen deposition on the distribution of immune cells, we further analyzed the spatial correlation between collagen deposition and CD8^+^ T cells infiltration. The results showed that abundant CD8^+^ T cells were present at sites of collagen deposition (Fig. 3F). Therefore, we analyzed the distribution of CD8^+^ T cells in two regions (tumor nest and stroma, including tumor margin and intratumor stroma). In tumor nest, CD8^+^ T cells were significantly less infiltrated in KL tumors, however, at the tumor margin where collagen deposited, CD8^+^ T cells infiltration were much higher in KL tumors (Fig. 3G).

The above results revealed that (i) compared with KP tumors, KL tumor had excessive ECM deposition; (ii) excessive collagen deposition blocked CD8^+^ T cells infiltrating into tumor nest to exert killing function; and (iii) CD8^+^ T cells could barely infiltrate into tumor nest owing to the collagen deposition and were accumulated in the margin of KL tumors.

### Activated CAFs were abundant in KL tumors and fibroblastic FAK is hyperactivated

**Fig. 4:**
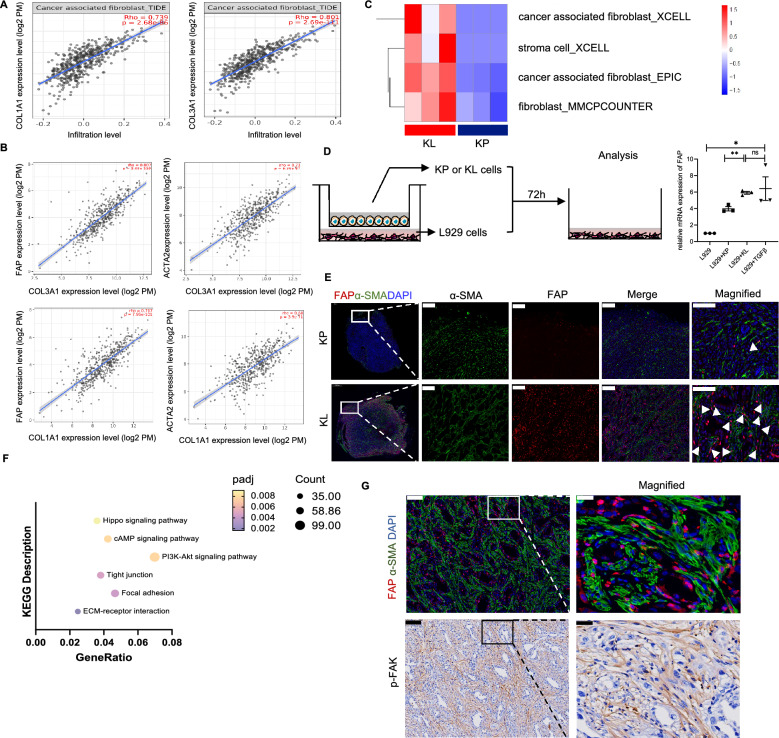
Activated CAF were abundant in KL tumors and fibroblastic FAK was hyperactivated. **A** The correlation between CAF infiltration and COL1A1 or COL3A1 via TIMER2.0. **B** The correlation between markers of activated CAF (FAP or α-SMA) and COL1A1 or COL3A1 via TIMER2.0. **C** Computed method calculating infiltrating levels of CAF in KP and KL subcutaneous tumors (n = 3 per each group) [48]. **D** Expression levels of FAP in L929 cells, after co-cultured with KL or KP tumors cells for 72 h by RT-qPCR. The gene expression data of 72 h treated L929 cells were normalized to the DMEM control group. Additional TGF-β was used as positive control for activating CAF. Data represents mean ± SEM from three independent experiments. *p < 0.05, **p < 0.01, ***p < 0.001, and ns represents p-values with no statistical difference. **E** Representative immunofluorescent staining of sections from KL or KP tumors. Red, FAP staining; green, α-SMA staining; blue, DAPI staining. Scale bars, 200 μm. magnified scale bars,100 μm. **F** KEGG analysis for significantly upregulated genes in KL tumors compared to KP tumors. Representative and related KEGG signal pathways among top 30 upregulated pathways were listed here. **G** Immunofluorescence staining and IHC staining co-localizing CAF and activated FAK. Scale bars, 100 μm. magnified scale bars, 20 μm. KL, *KRAS*^*G12D*^*LKB1*^*−/−*^; KP, *KRAS*^*G12D*^*TP53*^*−/−*^; CAF, cancer-associated fibroblast; FAK, focal adhesion kinase; FAP, fibroblast activation protein; SMA, smooth muscle actin; IHC, immunohistochemistry; TGF-β, transforming growth factor-beta; DMEM, dulbecco's modified eagle's medium; SEM, standard error of mean

**Fig. 5:**
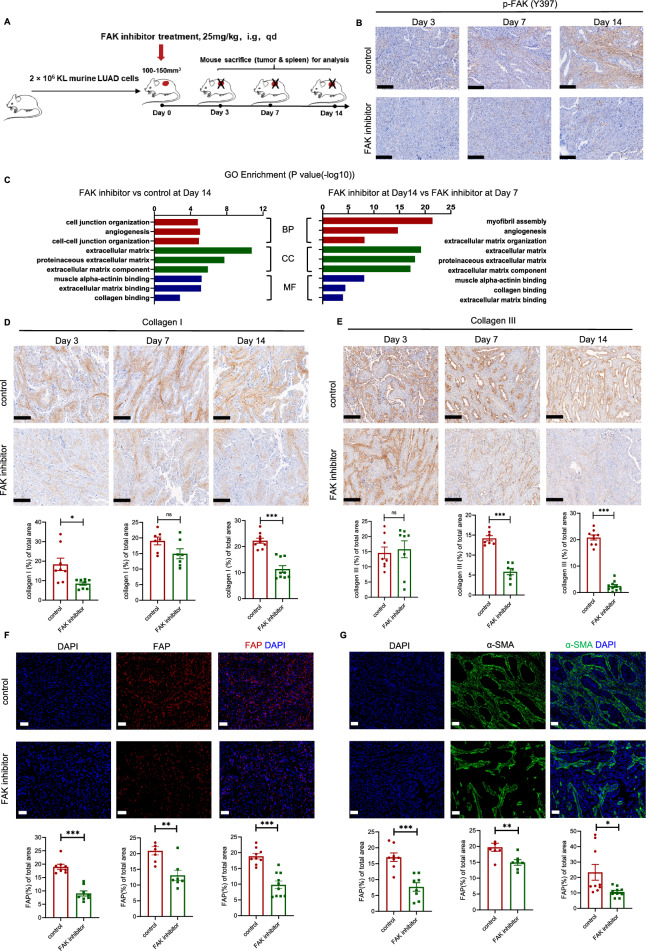
FAK inhibitor decreased collagen deposition and inhibited CAF activation. **A** Diagram depicting treated time schedule with FAK inhibitors for the subcutaneous KL tumor model. **B** IHC staining for p-FAK in KL tumors treated with FAK inhibitor at different time points (Day 3, 7,14). Scale bars, 200 μm. **C** GO analysis for significantly downregulated genes in KL tumors treated with FAK inhibitors at Day14 compared to control (left) and treated with FAK inhibitors at Day14 compared to Day7. Representative IHC or immunofluorescent staining and quantification of **D** Collagen I, scale bars, 200 μm. **E** Collagen III, scale bars, 200 μm. **F** FAP, scale bars, 50 μm. **G** α-SMA, scale bars, 50 μm, in KL tumors treated with FAK inhibitor at different time points (Day 3, 7,14). Red, FAP staining; green, α-SMA staining; blue, DAPI staining. Unpaired Student’s *t*-test was performed and results in each group were presented as mean ± SEM. *p < 0.05, **p < 0.01, ***p < 0.001, and ^ns^p-values with no statistical difference. KL, *KRAS*^*G12D*^*LKB1*^*−/−*^; CAF, cancer-associated fibroblast; FAK, focal adhesion kinase; FAP, fibroblast activation protein; SMA, smooth muscle actin; IHC, immunohistochemistry; BP, biological process; CC, cellular component; MF, molecular function; SEM, standard error of mean

CAFs are the predominant stromal cells in the TME [18]. Activated CAFs, which highly express α-SMA and fibroblast activating protein (FAP), usually accumulate at the edge of tumor nest, secret collagen fibers to surround the malignant cells with dense desmoplastic stroma, hence facilitate drug resistance via blocking anti-tumor drugs and immune cells into tumor nest [19, 20]. Therefore, we questioned whether CAFs were significantly activated in KL tumors. Firstly, using TIMER2.0 database (http://timer.cistrome.org/), we observed a positive correlation between the expression of dominant genes encoding collagen fibers and markers for activated CAFs (Fig. 4A and B). Consistently, our RNA-seq data also showed the more CAFs were infiltrated in KL tumors (Fig. 4C). To investigate whether KL tumor cells directly impacted the activation of fibroblasts, KL and KP tumor cells were co-cultured with mouse fibroblast (L929) in vitro. The results showed that co-culture with KL tumor cells significantly increased expression of FAP protein, suggesting that KL tumor cells could activate CAFs (Fig. 4D). Immunofluorescence further confirmed that activated CAFs were highly infiltrated in KL tumors (Fig. 4E). Next, we performed KEGG pathway analysis of dissected KL and KP tumors to explore the mechanisms of abundant activated CAFs. Interestingly, focal adhesions-related genes were enriched in KL tumors (Fig. 4F). Focal adhesions are integrated points of cellular membrane which link to extracellular matrix via integrin heterodimers [21, 22]. FAK is recruited to the sites of focal adhesions and get auto-phosphorylated upon ECM-induced integrin receptor activation [23]. Previous studies demonstrated that FAK activation was closely associated with stromal fibrosis and immunosuppressive TME [24, 25]. Further co-localization analysis in tumor specimens demonstrated that FAK was hyperactivated in CAFs (Fig. 4G). Given the fact that FAK activation within pancreatic cancer CAFs was associated with ECM synthesis and deposition [26], we studied whether fibroblastic FAK hyperactivation could lead to CAFs activation and collagen deposition. KL tumors-bearing mouse were treated with FAK inhibitor (IN10018, InxMed, 25 mg/kg) or natrosol as control and tumor components were assessed at different time points (Day 3, Day 7, Day 14) (Fig. 5A). As indicated, FAK inhibitor effectively inhibited FAK phosphorylation (Fig. 5B). Further GO analysis revealed that FAK inhibitor had significant impact on ECM-related genes (FAK inhibitor vs control at Day 14, FAK inhibitor at Day14 vs FAK inhibitor at Day 7) (Fig. 5C). Immunofluorescence and IHC results also demonstrated FAK inhibitor could significantly decrease fibrosis and CAFs activation (Fig. 5D-G).

Angiogenesis is one of the hallmarks of cancer and blood vessels are also the important components of the TME [11, 27]. Notably, previous evidence showed that FAK pathway was associated with angiogenesis and tumor growth [28–30]. Therefore, we questioned whether the use of FAK inhibitor has an impact on TME via inhibiting tumor angiogenesis in KL tumors. Indeed, GO analysis and immunofluorescence staining revealed that angiogenesis-associated genes were highly enriched in KL tumors (Additional file 4: Fig. S2A, B). Although FAK inhibitor slightly alleviated hypoxia at Day 7, FAK inhibitor had no significant impact on angiogenesis (Additional file 4: Fig. S2C, D).

In summary, these findings suggested that (i) abundant activated CAFs resulted in excessive collagen deposition in KL tumors; and (ii) fibroblastic FAK activity contributed CAFs activation and hence, induced collagen formation.

### Inhibition of FAK suppressed tumor progression via boosting immune response in KL tumors

Therapeutic benefits of pharmacological FAK inhibition in previous preclinical models have been confirmed to directly inhibit FAK expressed by neoplastic cells and thus, reduced tumor growth, invasion and metastasis [31, 32]. To illustrate the cytotoxic effects of FAK inhibition on tumor cells, we utilized human NSCLC cell lines with *LKB1*-mutant (A549), *LKB1*-WT (H1299), mouse lung cancer cell line with *LKB1*-wild type (KP) and mouse lung cancer cell line with *LKB1*-loss (KL). However, knockdown of *LKB1* in LKB1-wild type cell lines and upregulation of LKB1 in *LKB1*-mutant or *LKB1*-loss cell lines did not significantly influence the activation of FAK pathway (Additional file 5: Fig. S3A). Additionally, FAK inhibitor had minimal impact on cell viability and no obvious effects on apoptosis of KL cells in vitro (Additional file 5: Fig. S3B, C). These results provided the evidence that FAK inhibitor did not directly exert cytotoxic effects on lung tumor cells.

**Fig. 6:**
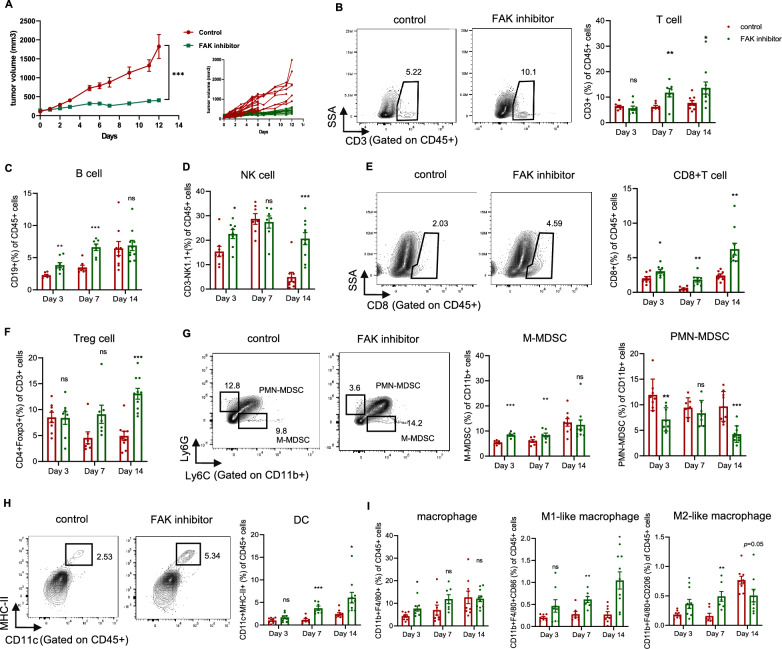
FAK inhibitor enhanced anti-tumor effect and mediated positive TME in KL tumors. **A** Volume change in mean (main) and individual (insert, “spider plots”) subcutaneous implanted tumors following treatment of FAK inhibitor (25 mg/kg) or control (0.5% Natrosol 250 HX) beginning at the day when implanted tumor volume reached 100-150mm^3^. Two-way analysis of variance (ANOVA) was performed. **B** Representative flow cytometry images at Day 7 and histogram showed the percentage of intratumoral CD3^+^ of CD45^+^ cells at different time points. Ratio of intratumoral **C** CD19^+^ cells, **D** CD3^−^NK1.1^+^ cells to CD45^+^cells at indicated time points. **E** Representative flow cytometry images at Day 14 and histogram showed the percentage of intratumoral CD8^+^ of CD45^+^ cells at indicated time points. **F** Ratio of CD4^+^Foxp3^+^ to CD3^+^ cells at indicated time points. **G** Representative flow cytometry images at Day 14 and histogram showed the percentage of intratumoral M-MDSC and PMN-MDSC at indicated time points. **H** Representative flow cytometry images at Day 14 and histogram showed the percentage of intratumoral CD11c^+^MHCII^+^of CD45^+^ cells at indicated time points. **I** Ratio of CD11b^+^F4/80^+^ cells and further quantification of macrophage subtypes (M1-like: CD86^+^, M2-like: CD206^+^) in two groups at indicated time points. Unpaired Student’s *t*-test was performed and results in each group were presented as mean ± SEM. *p < 0.05, **p < 0.01, ***p < 0.001, and ^ns^p-values with no statistical difference. KL, *KRAS*^*G12D*^*LKB1*^*−/−*^; TME, tumor microenvironment; FAK, focal adhesion kinase; NK, nature killer; MHC, major histocompatibility complex; Foxp3, forkhead box P3; M-MDSC, monocytic-myeloid-derived suppressor cells; PMN-MDSC, polymorphonucler-myeloid derived suppressor cells; SEM, standard error of mean

Next, we investigated the anti-tumor effect of FAK inhibition on tumor growth in KL mouse model and found that tumor growth was significantly attenuated (Fig. 6A). Therefore, we speculated FAK inhibitor might exert anti-tumor effect via remodeling the TME. Interestingly, FAK inhibitor significantly promoted proliferation of entire immune cells (CD45^+^Ki67^+^, Additional file 6: Fig. S4A). Moreover, FAK inhibitor significantly increased the proportions of T cells (on Day 7 and Day 14), B cells (on Day 3 and Day 7) and NK cells (on Day 3 and Day 14) (Fig. 6B–D). Specifically, a simultaneous increase in CD8^+^ T cells was observed with FAK inhibition on Day 3, Day 7 and Day 14 (Fig. 6E). Interestingly, on Day 14, more Foxp3^+^Tregs were also infiltrated in FAK inhibitor groups (Fig. 6F). Regarding myeloid cells, FAK inhibition facilitated more DCs infiltration on Day 7 and Day 14 (Fig. 6H). The quantity of M-MDSC were temporarily elevated with the treatment of FAK inhibitor on day 3 and day 7. In contrary, less PMN-MDSCs were infiltrated with FAK inhibition on day 14 (Fig. 6G). Although the total quantity of macrophages in two groups were not significantly altered, FAK inhibitor changed the proportions of macrophage phenotype, with more infiltration of M1-like macrophage and less infiltration of M2-like macrophage (Fig. 6I). The dynamic changes of immune cells in spleen were generally consistent with tumors upon FAK inhibition (Additional file 7: Fig. S5).

We also examined the dynamic change levels of related cytokines in tumor tissues. Firstly, RNA-seq demonstrated the corresponding changed immune cell-related cytokines, including CXCL9, CCL5, CCL2, VEGFA, CXCL5, CXCL1, CXCL2, CCL7 et al., were also significantly altered (Additional file 8: Fig. S6A). Specifically, CXCL1 was significantly reduced on Day 3 and Day 7 and CXCL2 was reduced on Day 7 with FAK inhibition, Meanwhile, CXCL9 was upregulated on Day 7 (Additional file 8: Fig. S6B).

The above findings suggested that FAK inhibitor effectively suppressed tumor growth via boost immune response, converting “immune-cold” tumors into “immune-hot” tumors. Upon FAK inhibition, more lymphocytes were infiltrated, especially CD8^+^ T cells. Meanwhile, several immunosuppressive cells, including PMN-MDSC and M2-like macrophage, were less infiltrated. Interestingly, FAK inhibitor could also upregulate PD-L1 expression on non-immune cells (CD45^−^PD-L1^+^, Additional file 6: Fig. S4B), implying a potential synergetic anti-tumor effect with anti-PD-1 antibody.

### Combining FAK inhibition and PD-1 blockade optimized anti-tumor effects in KL mouse models

**Fig. 7:**
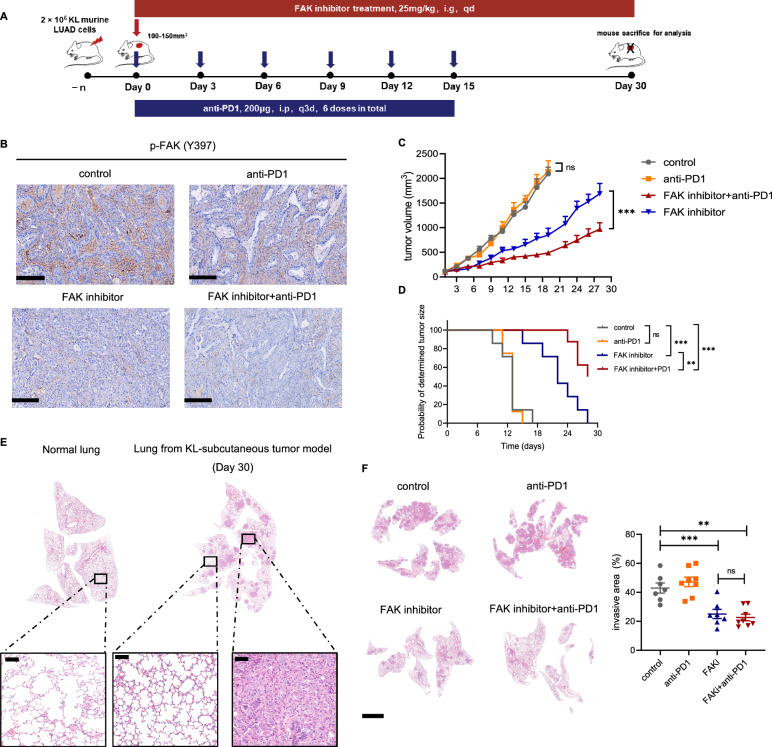
Combining FAK inhibitor and PD-1 blockade reduced tumor growth and lung metastasis. **A** Schematic diagram depicting treatment schedule for KL tumors. Red bar indicating the FAK inhibitor was daily administered. Blue bar indicating PD-1 blockade was administered every 3 days for 6 doses in total. Mouse were sacrificed for tumor analysis at indicated time. **B** IHC staining for p-FAK in each group. Scale bars, 200 μm. **C** Volume change in each group with different treatment schedule (n = 5–8 per group). Two-way analysis of variance (ANOVA) was performed. **D** Kaplan–Meier survival curves of the subcutaneous tumor-bearing mice treated as indicated since the day of tumor volume reached 100–150 mm^3^. Survival time of mice was defined from the day of tumor cell inoculation until the tumor volume reached 1000 mm^3^(n = 5–8 per group). **E** HE-stained normal lungs of C57 mice and lungs from KL tumor-bearing mice at Day30. Scale bars, 50 μm. **F** Representative images and quantification of invasive area in HE-stained lungs of KL tumors treated with different treatment schedules. Scale bars, 2 mm. Unpaired Student’s *t*-test was performed and results in each group were presented as mean ± SEM. *p < 0.05, **p < 0.01, ***p < 0.001, and ^ns^p-values with no statistical difference. FAK, focal adhesion kinase; PD-1, programmed death-1; IHC, immunohistochemistry; HE, hematoxylin–eosin; KL, *KRAS*^*G12D*^*LKB1*^*−/−*^; SEM, standard error of mean

**Fig. 8:**
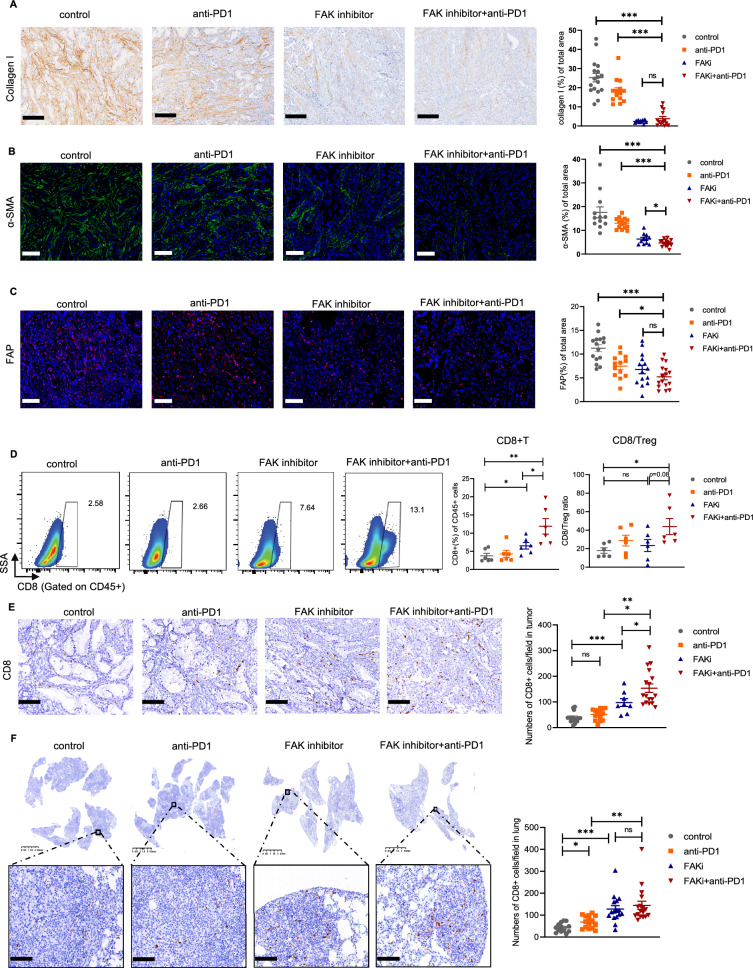
Impact of combining FAK inhibitor and PD-1 blockade on TME in KL mouse model. **A** Representative IHC staining of collagen I and quantification of collagen I staining area in different treatment schedule shown in Fig. 7A). Scale bars, 200 μm. **B** Representative IF staining of α-SMA and quantification of α-SMA staining area in different treatment schedule shown in Fig. 7A). Scale bars, 100 μm. **C** Representative IF staining of FAP and quantification of FAP staining area in different treatment schedule shown in Fig. 7A). Scale bars, 100 μm. **D** Quantification of CD8^+^TILs and ratio of CD8^+^T cells to Treg in each group. **E** Representative CD8 IHC staining and quantification of intratumoral primary tumor regions in each group. Scale bars, 100 μm. **F** Representative CD8 IHC staining and quantification of metastatic lung tumor regions in each group. Scale bars, 100 μm. Inset scale bars, 50 μm. Unpaired Student’s *t*-test was performed and results in each group were presented as mean ± SEM. *p < 0.05, **p < 0.01, ***p < 0.001, and ^ns^p-values with no statistical difference. FAK, focal adhesion kinase; PD-1, programmed death-1; TME, tumor microenvironment; IHC, immunohistochemistry; IF, immunofluorescence; FAP, fibroblast activation protein; TIL, tumor infiltrating lymphocyte; Treg, regulatory T cell; SEM, standard error of mean

Based on our previous findings, we further examined the anti-tumor effect of combining FAK inhibition and PD-1 blockade in KL mouse models. Tumor-bearing KL models and the following schedules were shown in Fig. 7A. PD-1 blockade had no impact on FAK phosphorylation (Fig. 7B). As expected, FAK inhibitor monotherapy or plus PD-1 blockade could both attenuate tumor growth compared with either PD-1 blockade group or control. Importantly, as compared with FAK inhibitor alone, the combination therapy resulted in a synergetic anti-tumor effect (Fig. 7C). Furthermore, the combination of FAK inhibitor and PD-1 blockade also led to prolonged time to reach endpoint (the time when tumor volume reached 1000 mm^3^) (Fig. 7D). Notably, on Day 30, histological analyses of lung tissues from subcutaneous mouse model showed that tumor cells had metastasized to lung, suggesting KL tumor cells were aggressive and invasive (Fig. 7E). Interestingly, FAK inhibitor with and without PD-1 blockade could also effectively decrease the metastatic area in lung tissue and inhibit lung metastases compared with control of anti-PD1 monotherapy group (Fig. 7F). As expected, FAK inhibitor and the combination therapy also significantly decreased collagen deposition and inhibited CAFs activation (Fig. 8A–C). Moreover, vessel density was similar among the four groups (Additional file 9: Fig. S7).

We then investigated the impact of different treatment regimens on the TME. The results showed that the proportions of T cells, specifically CD8^+^T cells were significantly increased in the combination group (Additional file 10: Fig. S8A and Fig. 8D). Additionally, CD8/Treg ratio was numerically elevated in the combination group compared with FAK inhibitor monotherapy group (p = 0.08) (Fig. 8D). Further IHC staining showed that FAK inhibitor in combination with PD-1 blockade could increase CD8^+^ T cells infiltration in both subcutaneous and lung metastatic tumor tissues (Fig. 8E and F). The proportions of B cells, NK cells and CD8^+^GZMB^+^ T cells were also significantly increased with FAK inhibition while there was no significant difference between FAK inhibitor monotherapy and the combination therapy (Additional file 10: Fig. S8B–D). In terms of Tregs, the combination therapy numerically reduced Tregs infiltration compared with FAK inhibitor monotherapy group (Additional file 10: Fig. S8E). Regarding myeloid cells, the combination group or FAK inhibitor significantly promoted DCs infiltration and reduce PMN-MDSC and macrophages infiltration (Additional file 10: Fig. S8F–H).

Collectively, these results demonstrated FAK inhibitor could overcome primary resistance of KL tumors to PD-1 blockade.

## Discussion

**Fig. 9:**
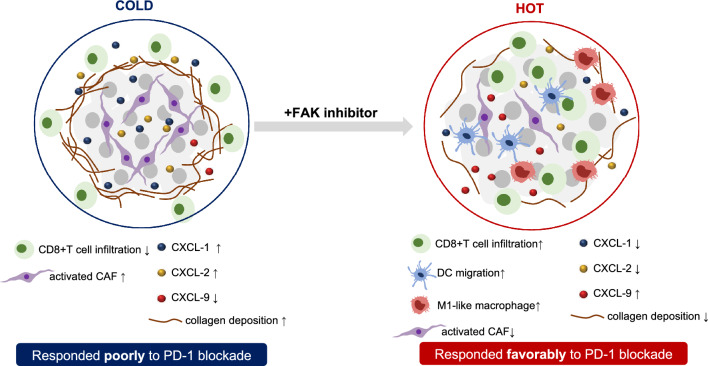
An overview schematic illustrating interactions between FAK inhibitor, tumor microenvironment and PD-1 blockade. Upon FAK inhibition, “immune-cold” KL tumors were converted into “immune-hot” tumors, with decreased collagen deposition and facilitated infiltration of anti-tumor immune cells, including CD8^ +^ T cells, DC cells and M1-like macrophages into tumors, additionally, CXCL1 and CXCL2 were downregulated and CXCL9 was upregulated, thus responded favorably to PD-1 blockade

The failure of PD-1 blockade therapy in KL lung adenocarcinoma emphasized the demand to uncover the characteristics of the TME in KL tumors and develop novel combinational therapeutic strategy. Utilizing KL and KP mouse model, we identified KL tumor as “immune-cold” tumor with excessive ECM collagen deposition that formed a physical barrier to block the infiltration of CD8^+^T cells. Mechanistically, abundant activated CAFs resulted from fibroblastic FAK activation contributed to the formation of the unique TME of KL tumors. Upon FAK inhibition to inhibit the activation of CAFs, our study demonstrated that FAK inhibitor could break the physical barrier via decreasing collagen deposition, and further facilitate the infiltration of CD8^+^ T cells, DC cells and M1-like macrophages into tumors, hence, convert “immune-cold” KL tumors into “immune-hot” tumors. The synergetic anti-tumor effect of FAK inhibitor and PD-1 blockade in KL mouse models provided new sights for developing effective combination strategies that reestablish antitumor immunity in KL tumors (Fig. 9).

It was reported that high stromal density could provide a barrier to the delivery of cytotoxic agents and has been postulated to limit T cells access to the tumor site in pancreatic cancer [33]. Similarly, in the preliminary exploration of the TME in KL lung adenocarcinoma, we found that dense collagen deposited and formed a physical barrier to prohibit the infiltration of immune cells to KL tumors. Also, a previous study demonstrated that the KL lung cancer model showed high levels of collagen deposition compared to *KRAS*-only model [12]. Owing to this physical barrier, the infiltration of immune cells, including CD8^+^ T cells, were generally decreased in KL tumors. Therefore, our findings might at least partially explain why KL tumors were not only refractory to ICI monotherapy, but also poorly responded to the combination of ICIs and chemotherapy [10].

Notably, not all immune cell infiltration was reduced in KL tumors. In our KL lung cancer mouse model, the infiltration of PMN-MDSC was significantly increased, therefore, the physical barrier cannot fully explain the formation of unique TME of KL tumors. Interestingly, CAFs, as the most abundant type of stromal cells in the TME, played a critical role in collagen formation and were hyperactivated in KL tumors. CAFs were not only the main source of collagen, but also secreted a variety of proteins, cytokines, chemokines to shape the TME [34]. Activated CAFs secreted factors such as IL-6, CCL2, and CXCL12 to affect T cell function [35]. CAFs could also secrete CXCL1 and CXCL2 to recruit suppressive immune cells PMN-MDSC into tumors [36]. Therefore, we believed that the abnormal activation of CAFs in KL tumors might be the key determiner to orchestrate the “immune-cold” tumors.

Focal adhesions are mediators of cell-ECM junctions that activate the intracellular FAK pathway [37]. In pancreatic cancer, the activation of FAK pathway of tumor cells can lead to extracellular fibrosis and immunosuppressive TME [33]. Also, FAK activation in squamous tumor cells increased the infiltration of Tregs via promoting the secretion of TGF-β and CCL5 and inhibiting the killing function of CD8^+^ T cells [38]. Previous studies have shown that LKB1 could inhibit the downstream signaling of FAK by phosphorylating MARK1, thereby inhibiting the formation of ECM collagen, and LKB1 deficiency can lead to the activation of the FAK pathway owing to the imbalance of oxidative stress levels [39, 40]. However, in our KL lung cancer model, LKB1 was not related to activation of FAK pathway in tumor cells. Meanwhile, fibroblastic FAK was hyperactivated and FAK inhibitor could significantly suppress the activation of CAFs and reduce the deposition of ECM collagen. There was evidence that the activation of intracellular FAK-Src pathway in CAFs could result in activation of fibroblasts by upregulating the expression of α-SMA [41]. Furthermore, in pancreatic cancer, inhibition of the FAK pathway in CAFs can effectively inhibit the activation of CAFs, thereby inhibiting tumor metastases, regulating the recruitment of immune cells, and promoting the degradation of extracellular collagen [26], which was consistent with our findings. However, Demircioglu et al. found that complete knockout of FAK protein in CAFs could facilitate the glycolysis of pancreatic cancer cells, thereby promoting the tumor growth [24]. Notably, the role of the intracellular FAK pathway depends on the helper of the scaffold protein and the activity of the kinase. Our current study used IN10018, a potent and highly selective ATP-competitive FAK inhibitor, to inhibit FAK activity but still retained FAK as a scaffold protein activity, representing a new option for inhibiting the activation of CAFs and remodeling the TME.

On the basis of FAK inhibitor could break the fibrotic barrier, we further demonstrated that FAK inhibitor could effectively suppress tumor growth via converting “cold” tumors into “hot” tumors, rather than cytotoxic effects directly on tumor cells. With FAK inhibition, more lymphocytes were infiltrated, especially CD8^+^T cells and DCs. Meanwhile, several immunosuppressive cells, including PMN-MDSC and M2-like macrophage, were less infiltrated. Zaghdoudi et al. also found that inhibition of FAK in CAFs could significantly promote the recruitment of M1-like macrophages and reduce the M2-like macrophages [26]. Besides a direct impact on the ECM collagen deposition, FAK inhibitor could also remodel the TME through regulating cytokines secretion, such as CXCL1 and CXCL2. Previous study showed that CAFs could mediate resistance to CSF-1R therapy by secreting CXCL1 and CXCL2 to recruit PMN-MDSCs, and the combined inhibition of CXCR2 and CSF-1R could overcome tumor resistance [36]. In addition, the level of CXCL9 was also elevated after application of FAK inhibitor in our study. CXCL9 is mainly secreted by M1-like macrophages and DCs to facilitate CD8^+^ T cells infiltration [42]. Therefore, we speculated that infiltrated DCs and M1-like macrophages upon FAK inhibition may further secrete CXCL9 to promote the infiltration of CD8^+^ T cells.

Previous study demonstrated that combing FAK inhibition and KRAS G12C inhibitors could improve the treatment outcomes for KRAS G12C mutant cancers through the regulation of the FAK-YAP signaling [43]. Given the fact that FAK inhibitor could remodel the TME in the current study, we next utilized KL mouse model to investigate the feasibility and efficacy of combining FAK inhibitor and PD-1 blockade. As expected, FAK inhibitor could overcome primary resistance of KL tumors to PD-1 blockade and a synergetic anti-tumor effect of FAK inhibitor and PD-1 blockade in KL tumors was observed. Notably, the combination therapy could also effectively inhibit lung metastases. Similarly, in PD-L1^+^ triple negative breast cancer cells lines, FAK inhibitor and PD-L1 antibody also exerted synergetic anti-tumor effects [44, 45]. Inhibition of the FAK pathway in CAFs could also significantly inhibited lung metastases in breast cancer [25].

Although the current study provided the new insight of therapeutic strategy for KL lung cancer tumors, however, several limitations and further investigations need to be acknowledged: (i) KP tumors responded well to PD-1 blockade, however, the impact of FAK inhibitor on KP tumors remained unclear. Peng et al. reported that collagen promoted anti-PD-1/PD-L1 acquired resistance in KP tumors [46]. Therefore, FAK inhibitor which could destroy stromal fibrosis might be a promising candidate in the treatment of KP tumor after its resistance to PD-1 blockade; (ii) Activated fibroblastic FAK was observed in KL tumors, however, how LKB1-deficiency KRAS-mutated tumor cells activated CAF and resulted in hyperactivating FAK pathway was not determined. It was reported that tumor-secreted LOXL-2 activated fibroblast through FAK signaling in breast cancer [41]. Secreted LOXL2 was also a novel therapeutic target that promoted gastric cancer metastasis via the FAK pathway [47]. Therefore, in KL tumors, whether LOXL-2 is a key regulator contributing to activated FAK pathway in CAF needs to be further investigated; and (iii) TME features in subcutaneous tumor models might be distinct from that in orthotopic tumors. Therefore, our findings should be verified in orthotopic mouse models in further investigations.

In summary, our findings emphasized the critical role of ECM collagen and CAFs in the formation of “immune-cold” KL tumors. FAK inhibition with a small molecular inhibitor could inhibit the activation of CAFs, destroy the stromal fibrosis barrier, and thus to convert the nature of KL tumors from “cold” to “hot”. Our study provided new sights for developing effective combination strategies that re-establish antitumor immunity in KL tumors in the future studies.

### Supplementary Information

**Additional file 1: Table S1.** Antibodies used in flow cytometry.

**Additional file 2: Table S2.** Names of collagen formation-related genes and top 20 collagen-related genes.

**Additional file 3: Figure S1.** The preparation and confirmation of cell lines in the study. **A** The schematic illustration for preparation of KL cell lines. **B** The confirmation of two cell lines by western blot analysis.

**Additional file 4: Figure S2.** Characterization of vessel density in TME. **A** GO analysis for significantly upregulated genes-related pathways in KL tumors compared to KP. **B** Representative immunofluorescent staining and quantification of CD31 in KL and KP tumors. Scale bars, 2 mm. Merge scale bars, 200 μm. Magnified scale bars, 100 μm. **C** Representative immunofluorescent staining and quantification of CD31 in KL tumors treated with FAK inhibitor at different time points (Day 3, 7,14). red, CD31 staining; blue, DAPI staining. Scale bars, 100 μm. **D** Representative IHC staining and quantification of HIF-1α in KL tumors treated with FAK inhibitor at different time points (Day 3, 7,14). Scale bar, 200um. Unpaired Student’s *t*-test was performed and results in each group were presented as mean ± SEM. *p < 0.05, **p < 0.01, ***p < 0.001, and ^ns^p-values with no statistical difference.

**Additional file 5: Figure S3.** The intrinsic interactions between LKB1 and FAK pathway in tumor cells. **A** Western blot analysis for FAK signaling upon overexpressing or interfering with LKB1 in three cell lines. **B** Cell viability tests for KL cell lines treated with FAK inhibitors for three time points. **C** Representative flow cytometry images and cell apoptosis analysis for KL tumor cells treated with different doses of FAK inhibitors and different time points. One-way analysis of variance (ANOVA) was performed. ns represents p-values with no statistical difference.

**Additional file 6: Figure S4.** FAK inhibitor enhanced proliferation of immune cells and elevated PD-L1 expression on tumor cell. **A** Representative flow cytometry images at Day 14 and histogram showed the percentage of intratumoral Ki67 + of CD45 + cells at indicated time points. **B** Representative flow cytometry images at Day 14 and histogram showed the percentage of intratumoral CD45^−^PD-L1^+^ of total viable cells at indicated time points. Unpaired Student’s *t*-test was performed and results in each group were presented as mean ± SEM. *p < 0.05, **p < 0.01, ***p < 0.001, and ^ns^p-values with no statistical difference.

**Additional file 7: Figure S5.** FAK inhibitor mediated positive immune-response in spleen in KL tumor models. **A** Representative flow cytometry images at Day 14 and histogram showed the percentage of splenic CD3^+^ of CD45^+^ cells at different time points. Ratio of splenic, **B** CD19^+^ cells, **C** CD3^−^NK1.1^+^ cells to CD45^+^cells, **D** CD4^+^Foxp3^+^ to CD3^+^cells at indicated time points. **E** Gating strategy for subtypes of CD4^+^ or CD8^+^T cells in spleen (left) and histogram showed the percentage of splenic CD8^+^T cells, naïve CD8^+^T cells (CD44^low^CD62L^high^), effector memory CD8^+^T cells (CD44^high^CD62L^low^), central memory CD8^+^T cells (CD44^high^CD62L^high^), CD4^+^T cells, naïve CD4^+^T cells (CD44^low^CD62L^high^), effector memory CD4^+^T cells (CD44^high^CD62L^low^), central memory CD4^+^T cells (CD44^high^CD62L^high^). **F** Gating strategy for DCs and histogram showed the percentage of splenic CD11c^+^MHC-II^med^ DCs and CD11c^+^MHC-II^high^ DCs of CD45^+^ cells at different time points. **G** Gating strategy for MDSC subtypes and histogram showed the percentage of splenic M-MDSC (CD11b^+^Ly6C^high^Ly6G^low^) and PMN-MDSC (CD11b + Ly6C^low^Ly6G^high^) at different time points. **H** Representative flow cytometry images at Day 7 and histogram showed the percentage of splenic macrophage (CD11b^+^F4/80^+^) of CD45 + cells at different time points. Unpaired Student’s *t*-test was performed and results in each group were presented as mean ± SEM. *p < 0.05, **p < 0.01, ***p < 0.001, and ^ns^p-values with no statistical difference.

**Additional file 8: Figure S6.** Cytokines in KP, KL tumors and impact of FAK inhibitors on levels of cytokines in KL tumors. **A** Heatmap of RNA-seq showing cytokines-related genes statistically significantly (FDR < 0.05) differentially expressed in KL subcutaneous tumors. **B** Luminex liquid suspension chip evaluating indicated cytokines levels upon FAK inhibition at different time points (D3, D7). Unpaired Student’s *t*-test was performed and results in each group were presented as mean ± SEM. *p < 0.05, **p < 0.01, ***p < 0.001, and ns represents p-values with no statistical difference.

**Additional file 9: Figure S7.** Impact of combining FAK inhibitor and PD-1 blockade on vessel density in KL mouse model. Representative immunofluorescent staining and quantification of CD31 in KL tumors treated with different treatment schedule. red, CD31 staining; blue, DAPI staining. Scale bars, 100 μm. One-way analysis of variance (ANOVA) was performed. ns represents p-values with no statistical difference.

**Additional file 10: Figure S8.** Impact of combining FAK inhibitor and PD-1 blockade on TME in KL mouse model. Ratio of **A** CD3 + , **B** CD3 + CD19 + , **C** CD3-NK1.1 + , **D** CD8 + GZMB + to CD45 + cells, **E** CD4 + Foxp3 + to CD3 + cells. **F** Representative flow cytometry images and histogram showed the percentage of intratumoral DCs in each group. **G** Representative flow cytometry images and histogram showed the percentage of M-MDSC and PMN-MDSC in each group. **H** Representative flow cytometry images and histogram showed the percentage of macrophage, M1-like macrophage and M2-like macrophage in each group. Unpaired Student’s *t*-test was performed and results in each group were presented as mean ± SEM. *p < 0.05, **p < 0.01, ***p < 0.001, and ^ns^p-values with no statistical difference.

## Data Availability

The OAK and POPLAR trials were available as supplementary information in Gandara DR et al. The MSKCC studies are available at www.cbioportal.org. The patient survival data and genetic aberrations were extracted for subgroup analysis. The datasets generated during and/or analyzed during the current study are available from the corresponding author on reasonable request.

## Abbreviations

NSCLC: Non-small cell lung cancer
TME: Tumor microenvironment
KL: *KRAS* ^*G12D*^ *LKB1* ^*−*^^*/*^^*−*^
KP: *KRAS* ^*G12D*^ *TP53* ^*−*^^*/*^^*−*^
LKB1: Liver kinase B1
FAK: Focal adhesion kinase
ECM: Extracellular matrix
CAF: Cancer-associated fibroblasts
ICIs: Immune checkpoint inhibitors
PD1: Programmed death-1
ORR: Objective response rate
DC: Dendritic cells
TANs: Tumor associated neutrophils
PFS: Progression-free survival
OS: Overall-survival
TAMs: Tumor-associated macrophage
GSEA: Gene set enrichment analysis
FAP: Fibroblast activating protein
NK: Nature killer
MHC: Major histocompatibility complex
Foxp3: Forkhead box P3
M-MDSC: Monocytic-myeloid-derived suppressor cells
PMN-MDSC: Polymorphonucler-myeloid derived suppressor cells
BP: Biological process
CC: Cellular component
MF: Molecular function
TIL: Tumor infiltrating lymphocyte
SMA: Smooth muscle actin
IHC: Immunohistochemistry
TGF-β: Transforming growth factor-beta
DMEM: Dulbecco’s modified eagle's medium
HE: Hematoxylin–eosin
Treg: Regulatory T cell
SEM: Standard error of mean
