# Supplementary material for: Targeting focal adhesion kinase boosts immune response in KRAS/LKB1 co-mutated lung adenocarcinoma via remodeling the tumor microenvironment
Source: Exp Hematol Oncol. 2024 Jan 30;13:11. doi: 10.1186/s40164-023-00471-6 (PMC10826079; doi:10.1186/s40164-023-00471-6)
Supplement: Supplementary file 1 — Additional file 1: Table S1. Antibodies used in flow cytometry. [file 40164_2023_471_MOESM1_ESM.docx]

| **Table S1. Antibodies used in flow cytometry** | | | | | | |
| --- | --- | --- | --- | --- | --- | --- |
| Antibody | Clone | Fluoroscence | Vendor | Identifier | Dilution |  |
| anti-mouse CD45 | 30-F11 | APC-Cy7 | BD Pharmingen | 557659 | 1:200 |  |
| anti-mouse CD11b | M1/70 | FITC | BD Pharmingen | 561688 | 1:500 |  |
| anti-mouse CD11c | HL3 | APC | BD Pharmingen | 561119 | 1:200 |  |
| anti-mouse F4/80 | T45-2342 | BV421 | BD Pharmingen | 565411 | 1:200 |  |
| anti-mouse CD86 | GL1 | PEcy7 | BD Pharmingen | 560582 | 1:200 |  |
| anti-mouse CD206 | MR6F3 | PE | eBioscience | 12-2061-80 | 1:200 |  |
| anti-mouse CD83 | Michel-19 | BV510 | BD Pharmingen | 563222 | 1:200 |  |
| anti-mouse MHCII | M5/114.15.2 | BV650 | BD Pharmingen | 563415 | 1:200 |  |
| anti-mouse Ly6C | AL-21 | BV605 | BD Pharmingen | 563011 | 1:200 |  |
| anti-mouse Ly6G | 1A8 | BB700 | BD Pharmingen | 566453 | 1:200 |  |
| anti-mouse CD3 | 145-2c11 | BV510 | BD Pharmingen | 563024 | 1:200 |  |
| anti-mouse CD19 | 1D3 | BV605 | BD Pharmingen | 563148 | 1:200 |  |
| anti-mouse NK1.1 | PK136 | PEcy7 | BD Pharmingen | 562062 | 1:200 |  |
| anti-mouse CD4 | RM4-5 | FITC | BD Pharmingen | 561835 | 1:500 |  |
| anti-mouse CD25 | PC61 | PE | BD Pharmingen | 561065 | 1:200 |  |
| anti-mouse Foxp3 | FJK-16s | APC | BD Pharmingen | 17-5773-80 | 1:200 |  |
| anti-mouse CD8a | 53-6.7 | PerCPCy5.5 | eBioscience | 561109 | 1:200 |  |
| anti-mouse CD279 | J43 | BV421 | BD Pharmingen | 565942 | 1:200 |  |
| anti-mouse CD274 | MIH5 | BV650 | BD Pharmingen | 740614 | 1:200 |  |
| anti-mouse granzymeB | GB11 | AF647 | BD Pharmingen | 515406 | 1:200 |  |
