# Supplementary material for: Targeting focal adhesion kinase boosts immune response in KRAS/LKB1 co-mutated lung adenocarcinoma via remodeling the tumor microenvironment
Source: Exp Hematol Oncol. 2024 Jan 30;13:11. doi: 10.1186/s40164-023-00471-6 (PMC10826079; doi:10.1186/s40164-023-00471-6)
Supplement: Supplementary file 2 — Additional file 2: Table S2. Names of collagen formation-related genes and top 20 collagen-related genes. [file 40164_2023_471_MOESM2_ESM.docx]

**Table S2. Names of collagen formation-related genes and top 20 collagen-related genes**

| **Collagen formation-related genes** | |
| --- | --- |
| Gene names | Col20a1, Cd151, Itga6, Dst, Col4a6, Col4a5, Col24a1, Adamts2, Col7a1, P3h2, P4ha3, Col23a1, Col27a1, Col5a3, Mmp9, Pxdn, Adamts14, P3h1, Col26a1, Lox, Col1a1, Col6a2, Col6a3, Col6a1, Col3a1, Col5a1, Col12a1, Loxl1, Col5a2, Bmp1, Loxl2, P3h3, Col1a2, Col16a1, P4ha2, Col8a1, Mmp7, Col10a1, P4hb, Serpinh1, Plod1, Tll1, Itgb4, Col4a2, Col4a1, Colgalt2, Lama3, Col13a1, Col4a4, Colgalt1, Col4a3, Loxl3, Lamc2, Lamb3 |
| **Top 20 collagen-related genes** | |
| Gene names | Itga6, Col4a6, Col4a5, Adamts2, Col6a2, Col6a3, Col3a1, Col5a1, Col12a1, Bmp1, Loxl2, Loxl1, Lox, Col1a1, P3h3, Col1a2, Col16a1, Col13a1, Lama3, Col4a4 |
