# Supplementary figures and images for: Targeting focal adhesion kinase boosts immune response in KRAS/LKB1 co-mutated lung adenocarcinoma via remodeling the tumor microenvironment
Source: Exp Hematol Oncol. 2024 Jan 30;13:11. doi: 10.1186/s40164-023-00471-6 (PMC10826079; doi:10.1186/s40164-023-00471-6)

**A**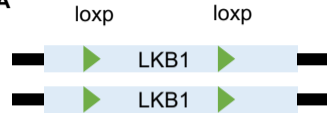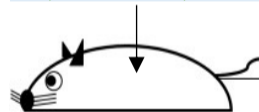 $KRAS^{G12D}LKB1^{loxp/loxp}$ 

Ad-Cre

intra-nasal

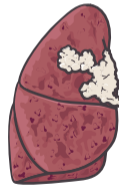

dissect

sorting

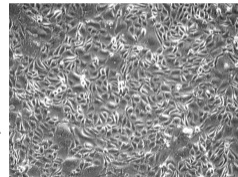 $KRAS^{G12D}LKB1^{-/-}$ **B**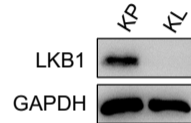

Supplement: Supplementary file 3 — Additional file 3: Figure S1. The preparation and confirmation of cell lines in the study. A The schematic illustration for preparation of KL cell lines. B The confirmation of two cell lines by western blot analysis. [file 40164_2023_471_MOESM3_ESM.pdf]

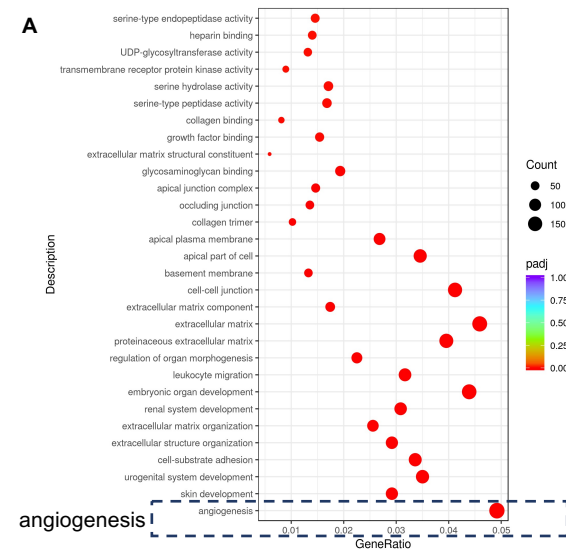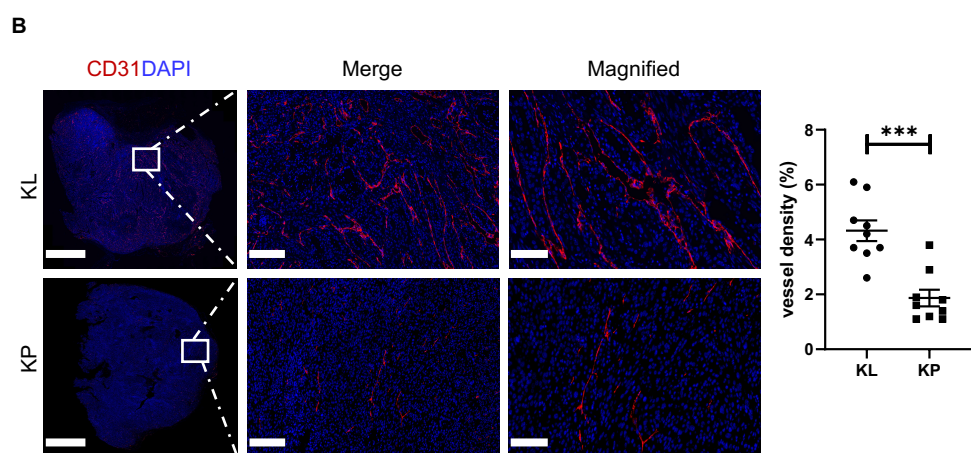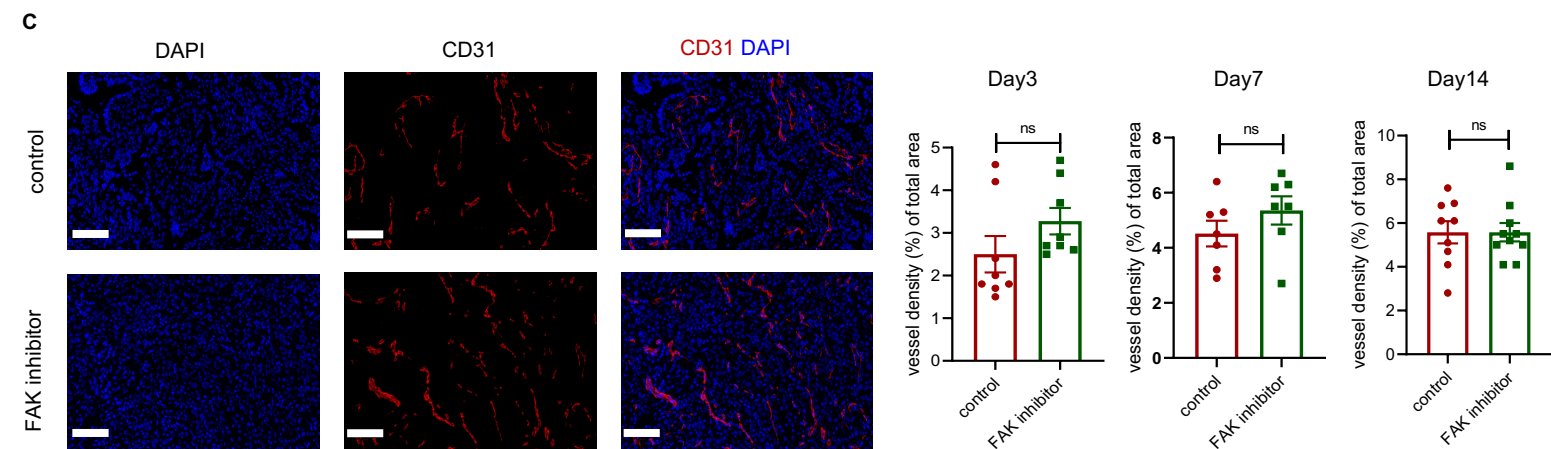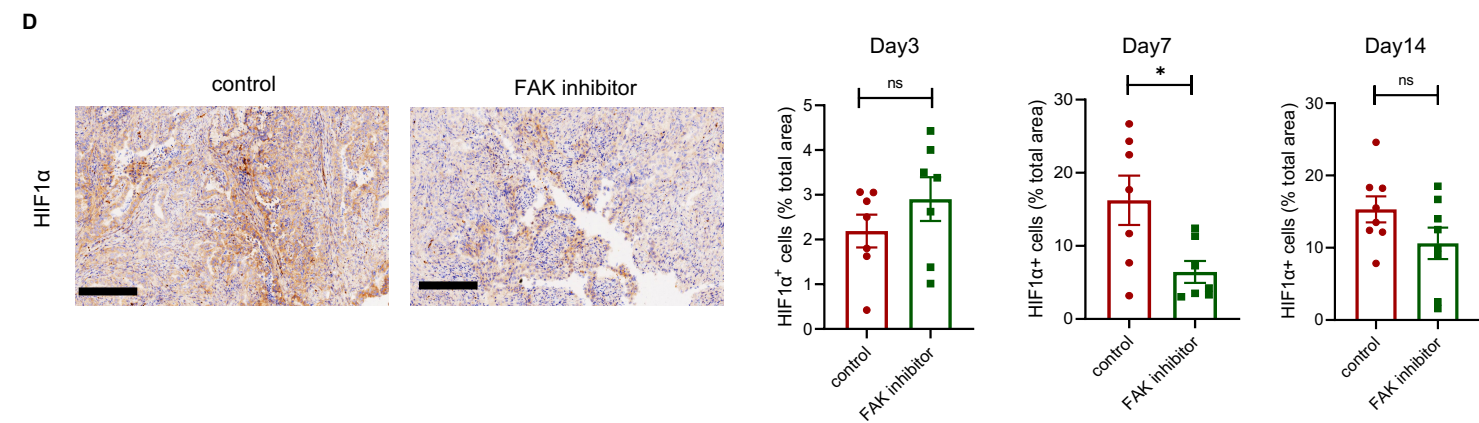

Supplement: Supplementary file 4 — Additional file 4: Figure S2. Characterization of vessel density in TME. A GO analysis for significantly upregulated genes-related pathways in KL tumors compared to KP. B Representative immunofluorescent staining and quantification of CD31 in KL and KP tumors. Scale bars, 2 mm. Merge scale bars, 200 μm. Magnified scale bars, 100 μm. C Representative immunofluorescent staining and quantification of CD31 in KL tumors treated with FAK inhibitor at different time points (Day 3, 7,14). red, CD31 staining; blue, DAPI staining. Scale bars, 100 μm. D Representative IHC staining and quantification of HIF-1α in KL tumors treated with FAK inhibitor at different time points (Day 3, 7,14). Scale bar, 200um. Unpaired Student’s t-test was performed and results in each group were presented as mean ± SEM. *p < 0.05, **p < 0.01, ***p < 0.001, and nsp-values with no statistical difference. [file 40164_2023_471_MOESM4_ESM.pdf]

**A**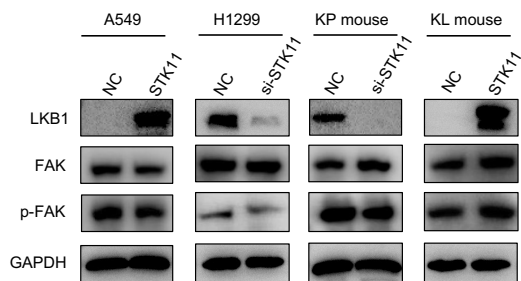**B**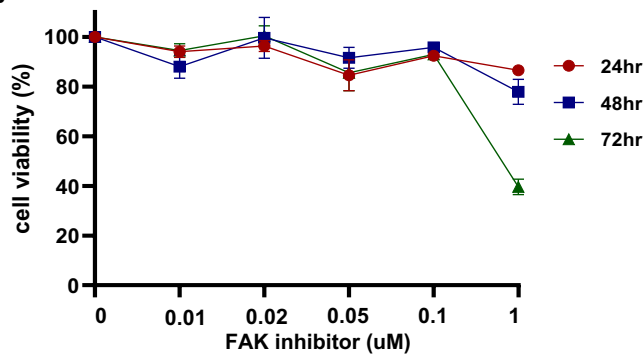**C**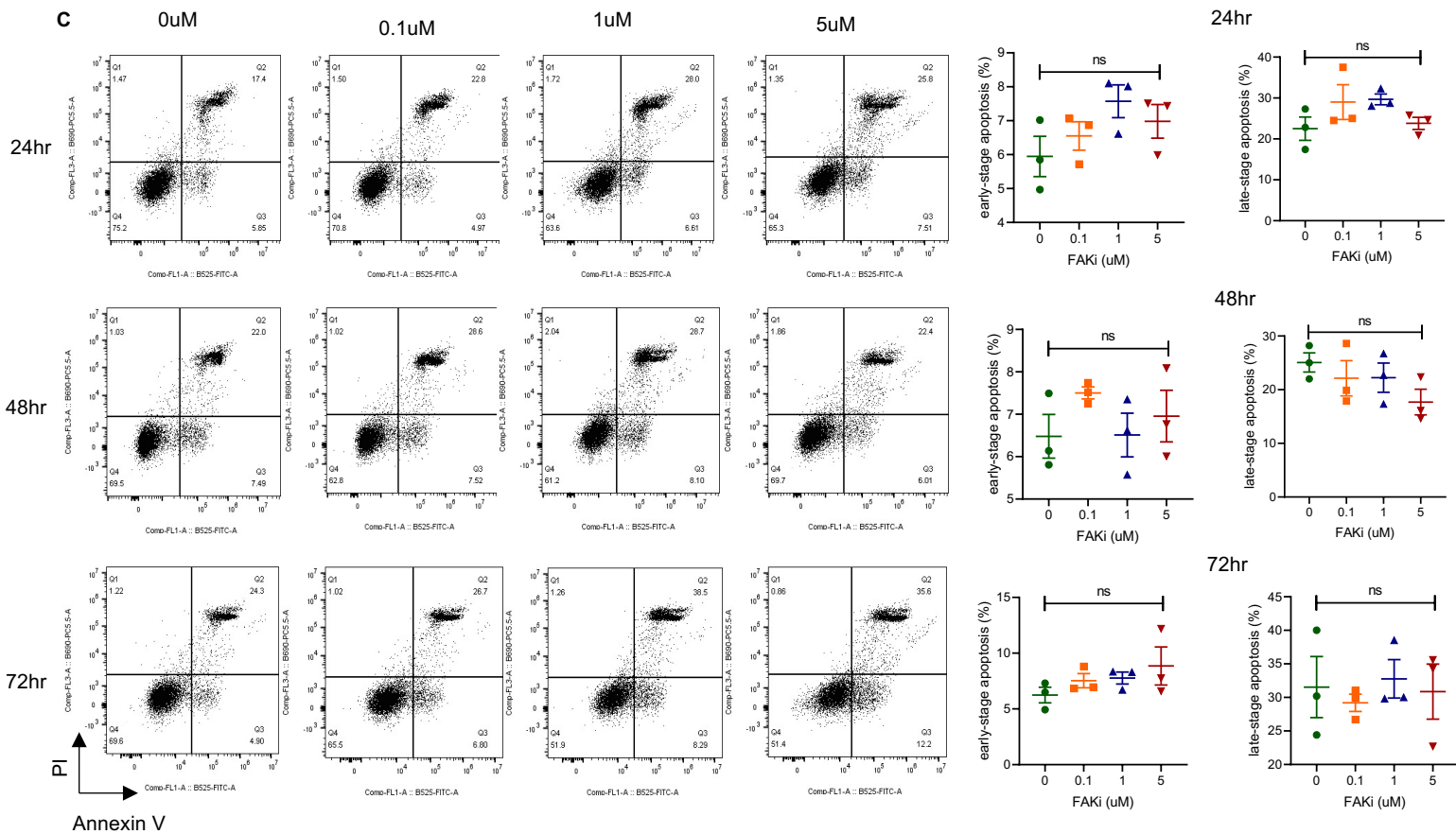

Supplement: Supplementary file 5 — Additional file 5: Figure S3. The intrinsic interactions between LKB1 and FAK pathway in tumor cells. A Western blot analysis for FAK signaling upon overexpressing or interfering with LKB1 in three cell lines. B Cell viability tests for KL cell lines treated with FAK inhibitors for three time points. C Representative flow cytometry images and cell apoptosis analysis for KL tumor cells treated with different doses of FAK inhibitors and different time points. One-way analysis of variance (ANOVA) was performed. ns represents p-values with no statistical difference. [file 40164_2023_471_MOESM5_ESM.pdf]

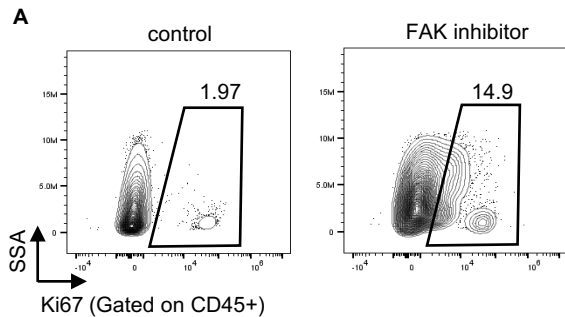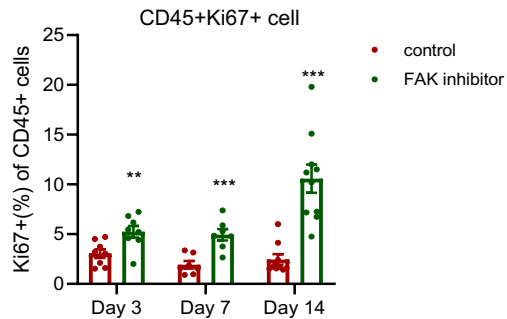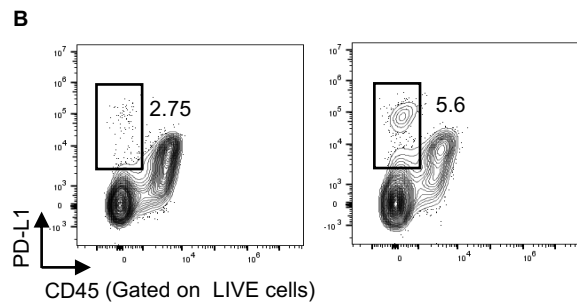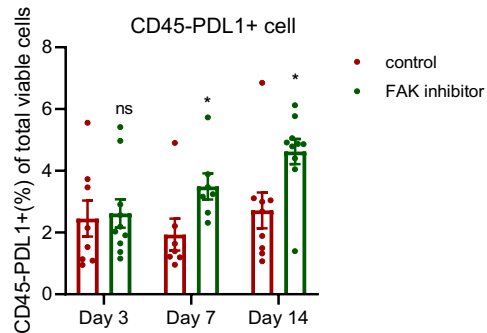

Supplement: Supplementary file 6 — Additional file 6: Figure S4. FAK inhibitor enhanced proliferation of immune cells and elevated PD-L1 expression on tumor cell. A Representative flow cytometry images at Day 14 and histogram showed the percentage of intratumoral Ki67 + of CD45 + cells at indicated time points. B Representative flow cytometry images at Day 14 and histogram showed the percentage of intratumoral CD45−PD-L1+ of total viable cells at indicated time points. Unpaired Student’s t-test was performed and results in each group were presented as mean ± SEM. *p < 0.05, **p < 0.01, ***p < 0.001, and nsp-values with no statistical difference. [file 40164_2023_471_MOESM6_ESM.pdf]

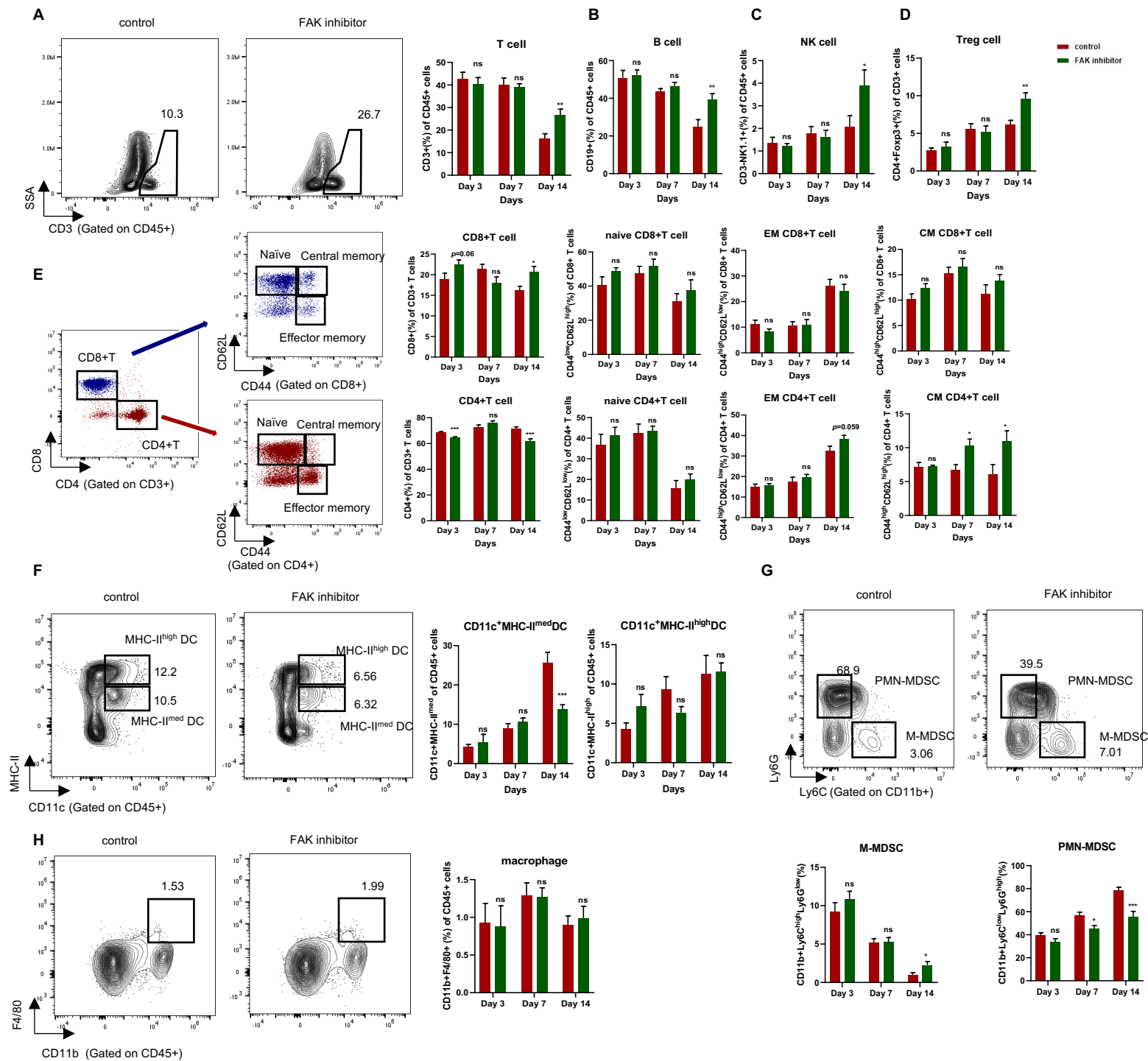

Supplement: Supplementary file 7 — Additional file 7: Figure S5. FAK inhibitor mediated positive immune-response in spleen in KL tumor models. A Representative flow cytometry images at Day 14 and histogram showed the percentage of splenic CD3+ of CD45+ cells at different time points. Ratio of splenic, B CD19+ cells, C CD3−NK1.1+ cells to CD45+cells, D CD4+Foxp3+ to CD3+cells at indicated time points. E Gating strategy for subtypes of CD4+ or CD8+T cells in spleen (left) and histogram showed the percentage of splenic CD8+T cells, naïve CD8+T cells (CD44lowCD62Lhigh), effector memory CD8+T cells (CD44highCD62Llow), central memory CD8+T cells (CD44highCD62Lhigh), CD4+T cells, naïve CD4+T cells (CD44lowCD62Lhigh), effector memory CD4+T cells (CD44highCD62Llow), central memory CD4+T cells (CD44highCD62Lhigh). F Gating strategy for DCs and histogram showed the percentage of splenic CD11c+MHC-IImed DCs and CD11c+MHC-IIhigh DCs of CD45+ cells at different time points. G Gating strategy for MDSC subtypes and histogram showed the percentage of splenic M-MDSC (CD11b+Ly6ChighLy6Glow) and PMN-MDSC (CD11b + Ly6ClowLy6Ghigh) at different time points. H Representative flow cytometry images at Day 7 and histogram showed the percentage of splenic macrophage (CD11b+F4/80+) of CD45 + cells at different time points. Unpaired Student’s t-test was performed and results in each group were presented as mean ± SEM. *p < 0.05, **p < 0.01, ***p < 0.001, and nsp-values with no statistical difference. [file 40164_2023_471_MOESM7_ESM.pdf]

**A**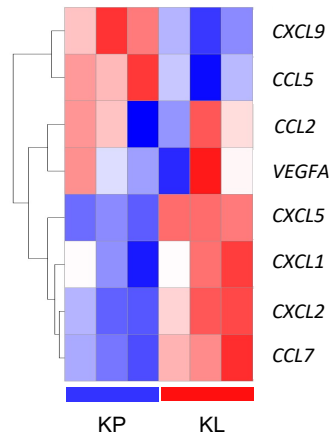**B**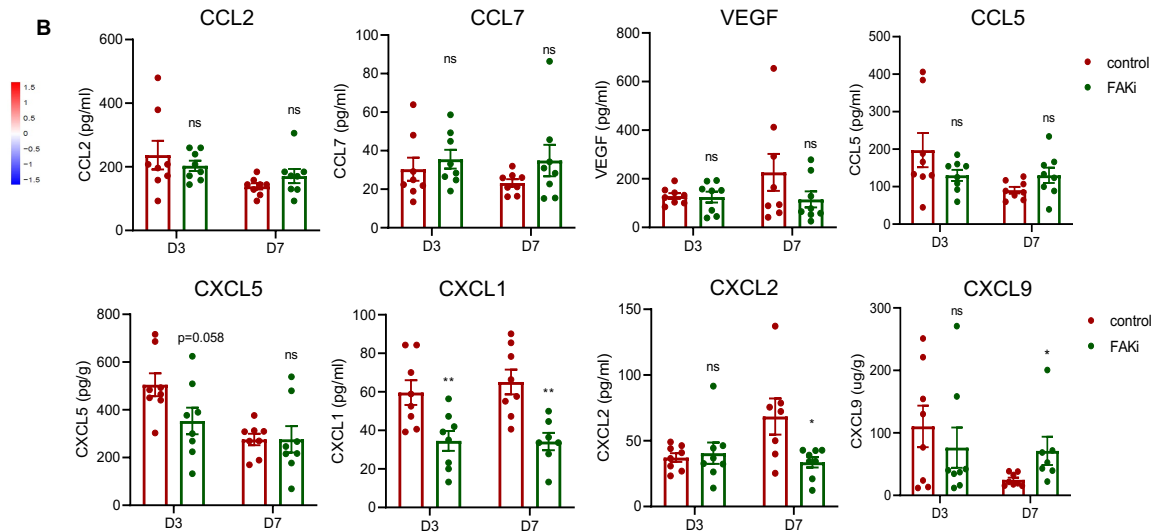

Supplement: Supplementary file 8 — Additional file 8: Figure S6. Cytokines in KP, KL tumors and impact of FAK inhibitors on levels of cytokines in KL tumors. A Heatmap of RNA-seq showing cytokines-related genes statistically significantly (FDR < 0.05) differentially expressed in KL subcutaneous tumors. B Luminex liquid suspension chip evaluating indicated cytokines levels upon FAK inhibition at different time points (D3, D7). Unpaired Student’s t-test was performed and results in each group were presented as mean ± SEM. *p < 0.05, **p < 0.01, ***p < 0.001, and ns represents p-values with no statistical difference. [file 40164_2023_471_MOESM8_ESM.pdf]

control

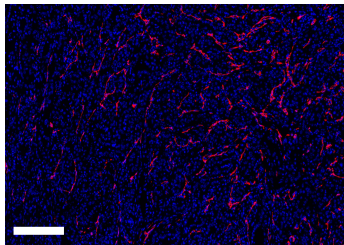

anti-PD1

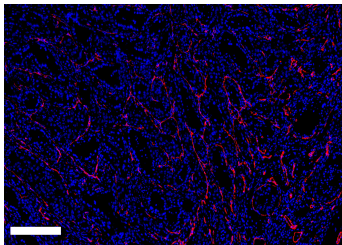

FAK inhibitor

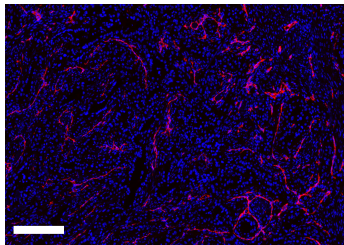

FAK inhibitor+anti-PD1

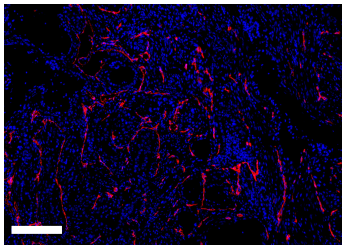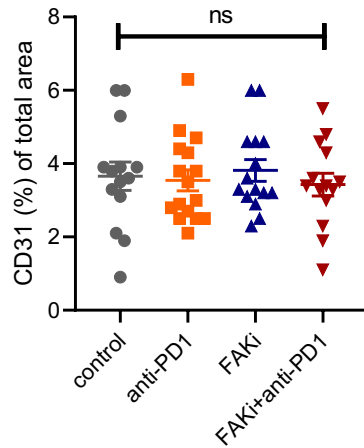

Supplement: Supplementary file 9 — Additional file 9: Figure S7. Impact of combining FAK inhibitor and PD-1 blockade on vessel density in KL mouse model. Representative immunofluorescent staining and quantification of CD31 in KL tumors treated with different treatment schedule. red, CD31 staining; blue, DAPI staining. Scale bars, 100 μm. One-way analysis of variance (ANOVA) was performed. ns represents p-values with no statistical difference. [file 40164_2023_471_MOESM9_ESM.pdf]

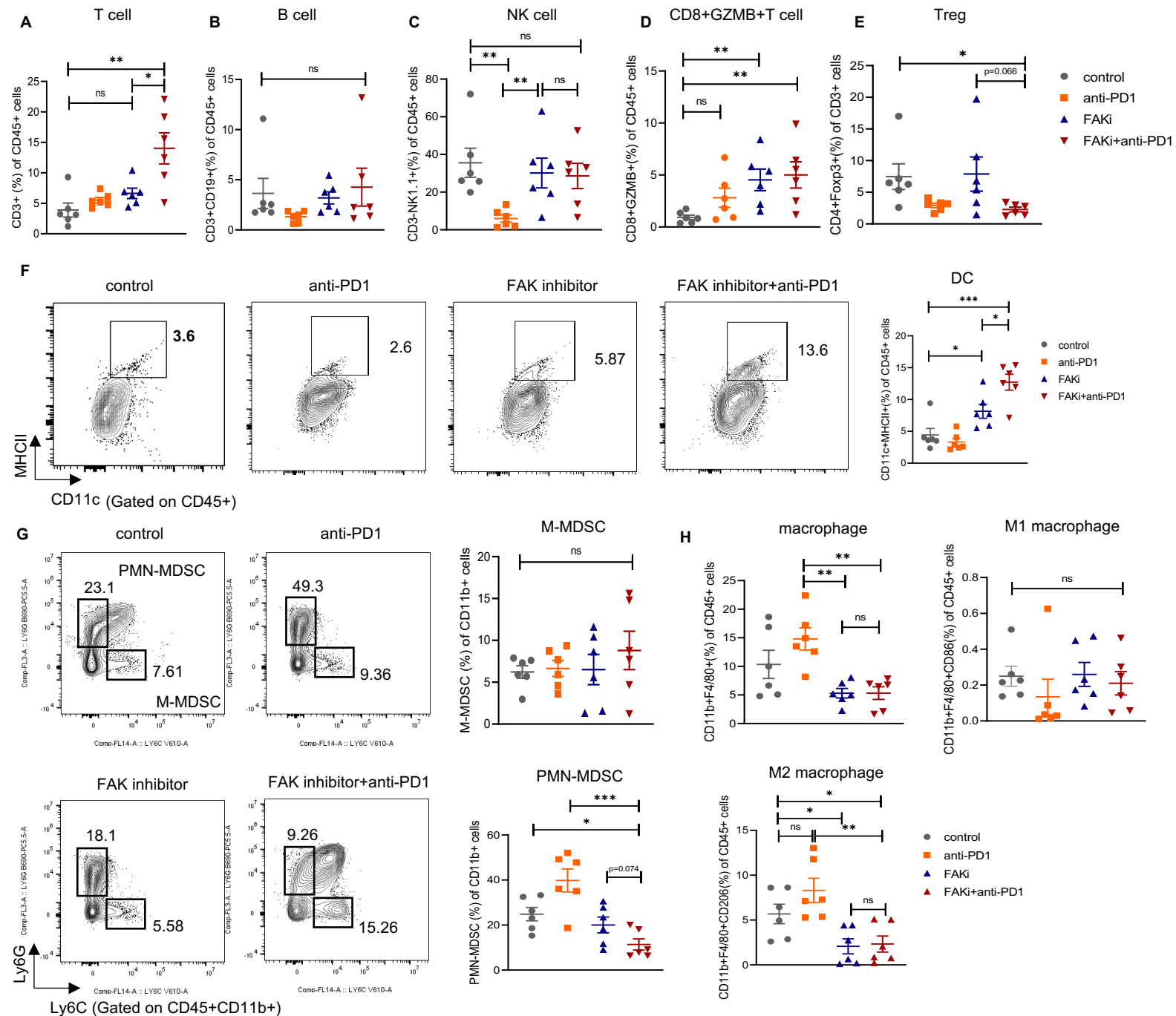

Supplement: Supplementary file 10 — Additional file 10: Figure S8. Impact of combining FAK inhibitor and PD-1 blockade on TME in KL mouse model. Ratio of A CD3 + , B CD3 + CD19 + , C CD3-NK1.1 + , D CD8 + GZMB + to CD45 + cells, E CD4 + Foxp3 + to CD3 + cells. F Representative flow cytometry images and histogram showed the percentage of intratumoral DCs in each group. G Representative flow cytometry images and histogram showed the percentage of M-MDSC and PMN-MDSC in each group. H Representative flow cytometry images and histogram showed the percentage of macrophage, M1-like macrophage and M2-like macrophage in each group. Unpaired Student’s t-test was performed and results in each group were presented as mean ± SEM. *p < 0.05, **p < 0.01, ***p < 0.001, and nsp-values with no statistical difference. [file 40164_2023_471_MOESM10_ESM.pdf]
